# Influences on the Uptake of and Engagement With Health and Well-Being Smartphone Apps: Systematic Review

**DOI:** 10.2196/17572

**Published:** 2020-05-29

**Authors:** Dorothy Szinay, Andy Jones, Tim Chadborn, Jamie Brown, Felix Naughton

**Affiliations:** 1 School of Health Sciences University of East Anglia Norwich United Kingdom; 2 Norwich Medical School University of East Anglia Norwich United Kingdom; 3 Behavioural Insights Public Health England London United Kingdom; 4 Department of Behavioural Science and Health University College London London United Kingdom

**Keywords:** mHealth, health app, engagement, uptake, systematic review, COM-B, TDF, digital health, mobile phone, smartphone, smartphone app

## Abstract

**Background:**

The public health impact of health and well-being digital interventions is dependent upon sufficient real-world uptake and engagement. Uptake is currently largely dependent on popularity indicators (eg, ranking and user ratings on app stores), which may not correspond with effectiveness, and rapid disengagement is common. Therefore, there is an urgent need to identify factors that influence uptake and engagement with health and well-being apps to inform new approaches that promote the effective use of such tools.

**Objective:**

This review aimed to understand what is known about influences on the uptake of and engagement with health and well-being smartphone apps among adults.

**Methods:**

We conducted a systematic review of quantitative, qualitative, and mixed methods studies. Studies conducted on adults were included if they focused on health and well-being smartphone apps reporting on uptake and engagement behavior. Studies identified through a systematic search in Medical Literature Analysis and Retrieval System Online, or MEDLARS Online (MEDLINE), EMBASE, Cumulative Index to Nursing and Allied Health Literature (CINAHL), PsychINFO, Scopus, Cochrane library databases, DataBase systems and Logic Programming (DBLP), and Association for Computing Machinery (ACM) Digital library were screened, with a proportion screened independently by 2 authors. Data synthesis and interpretation were undertaken using a deductive iterative process. External validity checking was undertaken by an independent researcher. A narrative synthesis of the findings was structured around the components of the capability, opportunity, motivation, behavior change model and the theoretical domains framework (TDF).

**Results:**

Of the 7640 identified studies, 41 were included in the review. Factors related to uptake (U), engagement (E), or both (B) were identified. Under *capability*, the main factors identified were app literacy skills (B), app awareness (U), available user guidance (B), health information (E), statistical information on progress (E), well-designed reminders (E), features to reduce cognitive load (E), and self-monitoring features (E). Availability at low cost (U), positive tone, and personalization (E) were identified as physical *opportunity* factors, whereas recommendations for health and well-being apps (U), embedded health professional support (E), and social networking (E) possibilities were social *opportunity* factors. Finally, the *motivation* factors included positive feedback (E), available rewards (E), goal setting (E), and the perceived utility of the app (E).

**Conclusions:**

Across a wide range of populations and behaviors, 26 factors relating to capability, opportunity, and motivation appear to influence the uptake of and engagement with health and well-being smartphone apps. Our recommendations may help app developers, health app portal developers, and policy makers in the optimization of health and well-being apps.

## Introduction

### Background

Digital behavior change interventions, such as smartphone apps, can be effective and cost-effective tools to change a range of health-related behaviors [1,2]. For example, there have been promising studies of apps, including (1) delivering health prevention messages for men who have sex with men [3], (2) self-managing diabetes [4] and cardiovascular diseases [5], (3) weight management [6-8], (4) alcohol reduction [9-11], (5) mental health interventions [12], and (6) managing long-term conditions [13]. For certain behaviors such as reduction of alcohol consumption, they could also address the barriers experienced by health professionals when delivering brief interventions in person, such as lack of necessary training [11] and to reduce the stigma associated with alcohol consumption [2]. The public health implications are substantial because of their potential to have a low incremental cost and broad reach.

Despite their promise, effect sizes reported in evaluations of app-based interventions are often small. One potential explanation is the level of uptake and engagement. Uptake refers to the act of downloading and installing a smartphone app. Engagement has been defined as “(1) the extent (e.g. amount, frequency, duration, depth) of usage and (2) a subjective experience characterized by attention, interest and affect” [14]. To date, low uptake and poor engagement are commonly observed with digital interventions, which are often insufficient to sustain behavior change [15,16]. However, there is a lack of evidence regarding the main factors contributing to this problem.

Systematic reviews that focused on one specific behavior or a certain type of health or well-being app suggest that the effectiveness of evidence-based smartphone apps can be improved by targeting the design and engagement features, such as user-friendly design, individualized and culturally tailored content, or health professional support [17-19]. A review based on experiential and behavioral perspectives conceptualized key factors that might affect engagement with digital behavior change interventions: the content (eg, behavior change techniques, social support, and reminders) and how the content is delivered (eg, professional support, personalization, and aesthetic features) [14].

To our knowledge, no systematic review that primarily seeks to identify factors that influence the uptake of and engagement with a wide range of health and well-being smartphone apps has yet been conducted. To narrow the focus of this review, the four public health priority behaviors related to prevention (smoking, alcohol consumption, physical activity, and diet) along with mental health and well-being were targeted.

### Theoretical Framework

The capability, opportunity, motivation, behavior (COM-B) model is a comprehensive framework that posits that individuals, to perform or change a behavior, need the capability to undertake it, the opportunity to take part in, and the motivation to engage with that behavior [20]. COM-B is increasingly being applied to inform the development of digital behavior change interventions [21-23]. The theoretical domains framework (TDF) [24] has previously been successfully applied for systematic reviews in other contexts [25,26]. The 14 domains of the TDF, described elsewhere [24], offer a concise coding framework that can be usefully conceptualized as possible targets for behavior change interventions. The TDF, being linked to the COM-B model [24], can be used as subthemes under the components of the COM-B model (see Multimedia Appendix 1).

### Objectives

This systematic review aimed to synthesize factors identified in studies that influence the uptake of and engagement with health and well-being smartphone apps among adults targeting public health priority behaviors (smoking, alcohol consumption, physical activity, and diet) and mental health and well-being, and mapped these factors under the components of the COM-B model and constructs of the TDF. This could help inform stakeholders in public health and policy makers, digital behavior change intervention developers, and providers of health and well-being smartphone app portals to better target uptake and engagement.

## Methods

### Systematic Review

The review was conducted according to the Preferred Reporting Items for Systematic Reviews and Meta-analyses (PRISMA; Multimedia Appendix 2) [27], and the protocol was registered on the International Prospective Register of Systematic Reviews (CRD42019120312). The review used a mixed methods approach to generate different but complementary knowledge about users’ views from qualitative findings and predictors and patterns of behavior from quantitative findings.

### Eligibility Criteria

Eligible studies had to explore factors that influence uptake or engagement with health and well-being smartphone apps among adults. Table 1 summarizes the inclusion and exclusion criteria using the Population, Intervention, Comparison or Context, Outcomes, and Study Type tool.

**Table 1:** List of inclusion/exclusion criteria.

PICOS^a^ component	Inclusion criteria	Exclusion criteria
Participants	Adults ≥18 years. Studies including individuals ≥16 years were included if at least 70% of the participants were ≥18 years	Apps targeting health professionals
Intervention and context	Studies investigating digital interventions using smartphone health and well-being behavior change apps on the following behaviors and outcomes: smoking, alcohol consumption, physical activity, diet and mental health, and well-being	Studies where the smartphone was not the primary intervention component
Outcomes	Qualitative: findings described as facilitators, barriers, determinants of uptake, or engagement with health or well-being apps (either already existing or planned to be developed), including perceptions, beliefs, experiences, and interest of the participants. Quantitative: uptake, measured as number of downloads, and engagement measured as number of logins, frequency of use, or any other relevant measure that tracks user engagement	Usability and user-testing studies, where functionality and app design were exclusively investigated for specific apps
Study design	All study designs were included	None

^a^PICOS: Population, Intervention, Comparison or Context, Outcomes, and Study Type.

### Search Strategy

#### Electronic Search

A systematic literature search was developed in consultation with a specialist librarian from the University of East Anglia and a senior information scientist from Public Health England (PHE). An iterative process helped to define the final search terms while ensuring a balance between sensitivity and specificity. A systematic literature search was performed in 8 electronic databases: Medical Literature Analysis and Retrieval System Online, or MEDLARS Online (MEDLINE), EMBASE, Cumulative Index to Nursing and Allied Health Literature (CINAHL), PsycINFO, Scopus, Cochrane library database, DataBase systems and Logic Programming (DBLP), and Association for Computing Machinery (ACM) Digital library. The databases were searched with no data limit, no publication or geographical restriction, but limited to the English language. Synonyms of 3 concepts were searched: (mhealth) AND (behavior change) AND (uptake or engagement; Multimedia Appendix 3 shows the MEDLINE search strategy). The electronic search was initially performed in November 2018 and was updated in August 2019.

#### Searching for Other Resources

Additionally, the search also included a manual search in key journals, such as *Journal of Medical Internet Research* and *Computers in Human Behavior*, and in *Google Scholar*. Reference lists of all included studies were hand-searched for additional studies. The search for gray literature included dissertations and theses, and unpublished research data and material were sought from government bodies and policy makers during stakeholder communication (PHE, National Health Service [NHS] in England).

### Identification of Studies

All records identified by the search strategy were exported to Endnote X9 and deduplicated. To reduce the likelihood of reviewer selection bias and to assess how reliably the study eligibility criteria were applied, a subsample (10%) of records was additionally screened by a second reviewer (FN) during the title and abstract screening. Interrater reliability based on the number of eligible and ineligible studies was tested using Cohen’s kappa statistics [28], with the following cut-offs being used: 0.41-0.60 to indicate moderate agreement, 0.61-0.80 substantial agreement, and 0.81-0.99 almost perfect agreement [28]. The full texts of potentially eligible studies were independently screened by DS, with 20% randomly selected and double-screened by FN. The exclusions of the studies were justified and recorded.

### Data Extraction

A data extraction proforma was developed by the first author following the existing Cochrane guidelines [29], and the subsequent data were extracted: study characteristics (author, date of publication, sample size and type, location of the study, type of app investigated in the study, aim of the study, methodological characteristics such as design, data collection, and participants), main findings related to the research question of this systematic review (including participants’ quotations and authors’ interpretations in the qualitative studies and reported results of the quantitative studies), and conclusions of each study. The data extraction was performed by 1 reviewer (DS) and was checked for accuracy by a second reviewer (FN).

### Quality Assessment

To assess the quality of the studies, critical appraisal was conducted using the latest version of the mixed methods appraisal tool (MMAT) [30]. MMAT is a unique tool [30] that was developed by pooling together the core relevant methodological criteria found in different well-known and widely used qualitative and quantitative critical appraisal tools [31-33].

The quality of all studies was assessed by the first reviewer (DS) and checked for accuracy by 2 other authors (FN and AJ). The tool is not intended to score the studies or to exclude papers but to offer a guide for interpreting findings [30].

### Data Synthesis and Analysis

Integrative synthesis was applied to analyze the data [34,35]. The focus of the synthesis was on interpreting the data using specific concepts of the TDF as a deductive coding framework, which, for ease of interpretation, is summarized under the components of the COM-B model. Using the integrated approach, the data were pooled together by findings viewed as answering the same research questions, rather than by methods (eg, quantitative vs qualitative) [34,35].

Deductive thematic synthesis, a methodology designed to enhance the transparency of synthesizing qualitative data [36], was used to conduct the data synthesis of the findings of the qualitative studies and the qualitative component of the mixed methods studies. Using line-by-line coding, the findings were coded deductively into the domains of the TDF. The coding was conducted by the first author, and a randomly selected 10% of the coding was checked for accuracy by another author (FN). Regular coding meetings were conducted to maintain consistency. The expert opinion of an independent researcher with extensive experience in systematic reviewing was sought for data synthesis. The integrative approach includes interpretation of the quantitative findings by *qualitizing* [35], which refers to the textual interpretation of the findings of the quantitative studies (regardless of the interpretation of the author) so they can be combined narratively with qualitative data [35].

## Results

### Included Studies

**Figure 1:**
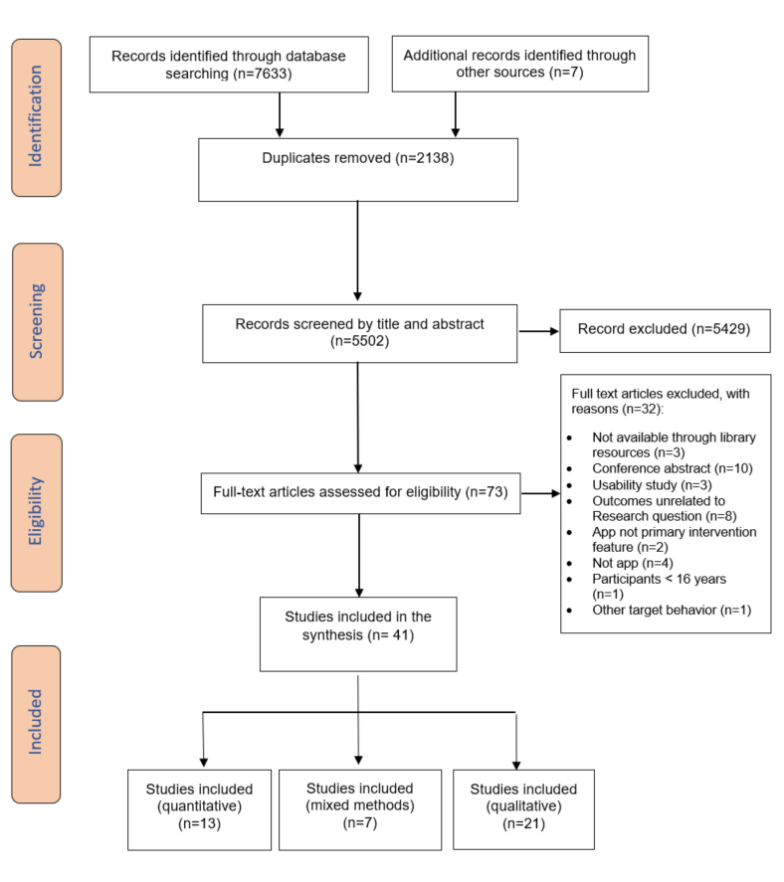
Preferred Reporting Items for Systematic Reviews and Meta-Analyses flowchart illustrating the inclusion and exclusion of studies.

A total of 7633 studies were initially retrieved, with a further 6 identified through manual search and reference check. An additional unpublished research report was received from stakeholders as part of the gray literature search process. No non-English papers were identified. A total of 2138 duplicates were removed. A total of 5429 studies were excluded based on the review of their titles and abstracts. Figure 1 illustrates the inclusion and exclusion of the studies following the guidance of the PRISMA flowchart [27].

During title and abstract screening, *substantial* agreement was achieved between the 2 independent reviewers (κ=0.63) [28]. Two types of disagreements were identified (one reviewer included studies that targeted app used in conjunction with a connected device and purely user-research studies) that limited agreement between the reviewers during the selection process, which were resolved through discussion and consultation with another author (AJ). After disagreements were resolved and the eligibility criteria were updated accordingly, 73 studies were identified as potentially meeting the inclusion criteria. All remaining titles and abstracts of records were assessed by 1 reviewer (DS). Of these, 41 studies were included in the review [37-77], out of which 13 were quantitative [41-44,49,53,55,63-65,68,76,77], 7 were mixed methods [38,47,59,62,73,74,78], and 21 were qualitative studies [37,39,40,45-47,50-52,54,56-58,60,61,66,67,70-72,75].

### Description of the Included Studies

The end users of the studies were described as the general public [37,39,42,44,46,47,50-54,56-59,65,71,72,75,76], college students [48], existing app users [38,43,46,49,55,63,67,77,78], male workers in the male-dominated industry [60], lesbian, gay, bisexual, transgender, queer, and other spectrum of sexuality and gender (LGBTQ+) communities [40], rural communities [57], Asian ethnic minorities [41], pregnant women [73], patients in primary care [45,61,74], adult cancer survivors [62], adults with diabetes [57], those infected with HIV [64], those with chronic disease [68], and those with a bipolar disorder [69]. The focus of some studies was very specific and targeted a certain health behavior or condition, including alcohol reduction [38,46,54,58,59,64], smoking cessation [40,58,67,72,77], increasing physical activity [39,45,48,49,53,62,65,68], weight management [47,48,51,53,63,65,66,71,78], depression [52,61], mindfulness [50], diabetes management [57], and health management in pregnancy [73]. Other studies were less specific and targeted a more general mental health app [43,60,70] and a more general health app [37,41,42,44,55,56,74-76]. In all, 15 studies investigated factors influencing one particular app [38,39,43,45,46,49,50,54,55,63,65,67,70,72,77]. The remaining 27 studies examined users’ perceptions of a wide range of apps or of a hypothetical app not yet developed.

The studies were published between 2011 and 2019 and were carried out in Australia [37,49,60,61,70], Belgium [69], Canada [40,51,55,67], China [68,73,76], Czech Republic [65], Ireland [45], Italy [39], New Zealand [47], Norway [75], Sweden [52], the United Kingdom [38,46,50,54,58,59,62,66,71,72,74], and the United States [41-44,48,53,56,57,63,64,77]. The study characteristics are summarized in Multimedia Appendix 4.

### Quality Assessment of the Studies Included

On the basis of MMAT [30], the majority of the studies employing qualitative methodology were deemed to be of high quality. Concerns related to the sample were identified across many quantitative studies. This included issues around sampling and lack of clarity as to whether the groups were comparable at baseline or whether the sample was representative of the general population. In 4 nonrandomized studies, confounders were not accounted for by the design and analysis. Out of 7 mixed methods studies, 2 were judged to be of low quality, out of which one is an unpublished report (gray literature) and the other one is a published short report. See Multimedia Appendix 5 for details of the quality assessment for each study.

### Data Analysis and Thematic Synthesis

Although not all the studies presented data for all aspects of this review, all studies presented some data that could be included in the synthesis. Evidence that was considered weakly explained or was judged to be unclear was not included in the summary of findings. An overview of the identified factors and the level of influence (uptake, engagement, or both) along with a brief description of each factor can be found in Table 2. Examples of supporting evidence are provided in the Textboxes 1-10.

**Table 2:** Factors identified in the systematic review.

COM-B^a^ component, TDF^b^ construct, and identified factor (source)^c^			Uptake, engagement, or both	Short description of the factor
**Physical capability**				
	**Skills**			
		App literacy [46,50,57,61,65]	Both	Technological competency
**Psychological capability**				
	**Knowledge**			
		App awareness [54,56,57,61,75]	Uptake	Knowledge of the existence of health and well-being apps
		User guidance [37,39,46,50,59,72]	Both	Instructions on how to effectively use the app
		Health information [47,51,53,54,56-58,62,69,72,75,78]	Engagement	Educational information related to health and well-being aspects
		Statistical information [37-39,46,52,54,57,66,67,71,72,75]	Engagement	A visual or numerical summary of progress
	**Memory, attention, and decision processes**			
		Well-designed reminders [37-40,43,46,48,51,52,54,56-58,62,66-69,71,78]	Engagement	The ability to customize reminders
		Less cognitive load [37,39,46,48,50,51,54,56-58,60,66,69,71,72,75]	Engagement	The app is not too time consuming, easy to use, and requires minimal input
		Coping games [40,60,67,72]	Engagement	Distraction activities within the app
	**Behavioral regulation**			
		Self-monitoring [36,38-40,45,48,51,52,55,57,59,60]	Engagement	The ability of the app to help self-regulation of the target behavior
		Established routines [38,48,50,54,66]	Engagement	Regularity in using the app
		Safety netting [37,61,66,73]	Engagement	Retaining the app for a potential precipitating event in the future
**Physical opportunity**				
	**Environmental context and resources**			
		Availability and accessibility [37,40,45,49,52,57,72,78]	Uptake	The ability to use a smartphone anytime anywhere
		Low cost [37,40,47,48,56,68,72,74]	Uptake	The price of the app
		Interactive and positive tone [46,51,57-60,69,71,72]	Engagement	Encouraging communication style
		Personalization to needs [37,38,40,47,50,52,56,57,60-62,69,71,72,75,78]	Engagement	The possibility to use an app that is tailored to a user’s needs
**Social opportunity**				
	**Social influences**			
		Recommendations [56-58,61,74]	Uptake	Suggestions received from other users
		Health practitioner support [37,40,51,52,57,59,62,67,69,72,73]	Engagement	Possibility to get in touch with health professionals and practitioners within the app
		Community networking [37,39,40,47,56,59,62,66-73,75]	Engagement	Social interaction with users with similar needs within the app or within their community
		Social media [39,40,48,54,56,58,61,66,67,71,72,75,78]	Engagement	A choice to connect to social media platforms
		Social competition [37,39,48,56,59,66,67]	Engagement	Competitive nature of the app with others or with themselves
		Personification of the app [39,45,47,48,50,56]	Engagement	Applying human attributes to the app
**Automatic motivation**				
	**Reinforcement**			
		Feedback [37,39,45-48,51,52,54,56,58,62,67,72]	Engagement	Feedback regarding the user’s performance
		Rewards [37,40,45,46,56-59,66,69,71,75]	Engagement	Tangible and intangible reward in response to the user’s effort
	**Emotions**			
		Curiosity [38,52,54,61]	Uptake	Desire to acquire knowledge and skills to use a behavior change tool
**Reflective motivation**				
	**Goals**			
		Goal setting [38,39,45,48,51,54,56,58,59,66,71,74]	Engagement	Establishing what the user would like to accomplish
	**Beliefs about consequences**			
		Perceived utility of the app [37,46,52,59,61,74]	Engagement	Discrepancy of what the users are looking for and what the app offers

^a^COM-B: capability, opportunity, motivation, behavior model.

^b^TDF: theoretical domains framework.

^c^Studies where the factors were identified.

### Physical Capability

#### Theoretical Domains Framework: Skills

Skills refer to one’s ability to perform an action and include constructs such as competencies, interpersonal skills, skill development, and practice (Textbox 1). App literacy [46,50,57,61,65], defined as technological competency to use a smartphone app, was reported by participants as being of high importance for both uptake and engagement. A basic level of app literacy is required to be able to download and initiate engagement with an app (see quote 1, Q1), whereas adequate app literacy skills would enhance users’ intentions to engage with an app (Q2) [46,50]. In a cross-sectional study, advanced app literacy was associated with increased use of the social functions of an app, such as networking, but not with the functions that target action planning and goal management [65]. This suggests that app literacy might be an important aspect for successful uptake, but this alone might not be enough to maintain engagement. In contrast, users have reported that lack of app literacy skills could trigger negative emotions toward themselves (eg, self-blame and disappointment of not being able to use an app) [46,50,61] and could contribute to their perceived low self-confidence in using technology [61].

**Illustrative quotes (Q1 and Q2) for factors mapped onto the physical capability subcomponent of the capability, opportunity, motivation, behavior model and coded under the theoretical domains framework: skills.**

*Uptake and engagement*

App literacy

- Quote 1: “I’d be happy to do it if I knew how to do it [but] I don’t know how to download apps...I need help with technology. Like, I’m 58 and I didn’t grow up in a technological age and so do find that I lack confidence with technology.” [61]
- Quote 2: “I’ve never used it [these apps] because I never got it to work the way I wanted it to.” [57]

### Psychological Capability

#### Theoretical Domains Framework: Knowledge

Multiple factors were identified under the TDF domain that covers rational, procedural, and other types of knowledge; information; and awareness of the existence of something (Textbox 2). App awareness [54,56,57,61,75], such as information on the existence of health and well-being apps, would positively influence the uptake of health and well-being smartphone apps (Q3). It was suggested that many participants were not aware of the availability of such tools, and some found the disorganized nature of the commercial app stores confusing and represented a barrier for uptake [61].

**Illustrative quotes (Q3-Q13) for factors mapped onto the psychological capability subcomponent of the capability, opportunity, motivation, behavior model and coded under the theoretical domains framework: knowledge.**

*Uptake*

App awareness

- Quote 3: “I didn’t realize that they had an app.” [57]

*Engagement*

User guidance

- Quote 4: “I want something to tell me ‘Do number 1 first, then number 2. When you’ve done this go here’ so I don’t have to think too much about it. Once I’ve got it up and running I’m fine.” [46]
- Quote 5: “Just at the beginning of the app, when you’ve downloaded it and you’re using it for the ﬁrst time, it should tell you what to do. But not every time. You don’t need guidance how to use it and where things are, because I think it would just be annoying.” [59]

Health information

- Quote 6: “[It is] important and really helps me to learn about bipolar disorder and read about stuff.” [67]
- Quote 7: “I... enjoy learning something new. It’s quite informative and makes you think about what you’re doing. [QG] helps you to understand a bit more about what’s going on...what could go wrong by continuing [to smoke].” [72]
- Quote 8: “I personally am scared of getting lymphedema, and still don’t know sometimes what exercises are good to prevent it, so I think that maybe educating people about [...] consequences of not exercising from a really good NHS source would be helpful.” [62]
- Quote 9: “I think everyone has heard that information many times. It’s actually quite patronizing...shallow stuff, not hard-hitting useful facts. It obviously isn’t a tailored app to each person, but it gives enough information that each person can relate to it in a tailored way. I find it really engaging, I suppose that’s why I stuck with it.” [72]

Statistical information

- Quote 10: “I like the numbers. I like to track stuff and have some figures behind it rather than just like, oh, I’ll go for a run today. I’ll be like, well, I’ll go for a run today but what’s my time from last time and how can I beat it? And I think that’s why this kind of app appeals to me. If I just put the drinks in and it just said you’re drinking too much but didn’t give any numbers behind it, I’d probably delete it within a few days.” [38]
- Quote 11: “It was like a visual of my day of smoking. And every day, you’d look at it, it went down and down and down, like it got better every day. So it was like a motivational thing to just look, like positive reinforcement.” [67]
- Quote 12: “I couldn’t find any graph that’s reflected the mood so therefore I didn’t see the point of having to fill that part out and I stopped filling it out.” [46]
- Quote 13: “If you're having a bad day or a couple of bad days, seeing it on [the app] as a reflection [of your bad days] just like kicks you in the face even more, you know?” [67]

User guidance [37,39,46,50,59,72], namely, instructions on how to effectively use an app, such as how to create achievable goals, influenced uptake and initial engagement. It was proposed that having a guide on how to use an app could positively affect the users’ intention to engage with it, and hence, users might be able to better regulate their behavior (Q4) [46,59]. However, the presence of a guide was reported off-putting and unnecessary for long-term engagement by producing negative emotions (eg, annoyance) once the knowledge regarding app functionality has been gathered (Q5) [59].

Available health information within the app was perceived by users as beneficial and positively influenced their engagement in several studies (Q6 and Q7) [47,51,53,54,56-58, 62,69,72,75,78]. Depending on the target behavior, end users wished to (1) access advice on exercise routines [39,56,62,66]; (2) seek nutritional education [39,51,56,57,66,70]; (3) widen their knowledge of health consequences [58,67,72]; (4) find out more about healthy living while living with a medical condition [62,73]; (5) know more about the conditions they are living with [69,73,75]; (6) improve their health literacy [75]; (7) demystify myths [72]; (8) receive health news updates, such as on smoking taxes and bans [72]; and (9) better understand alcohol units in the UK [54].

However, the quality of information was identified as potentially affecting engagement [72]. Some users wanted a credible source, a trustworthy and evidence-based guide with references to the information they receive (Q8) [62,70,73]. Health information that focuses on negative aspects of past behavior that cannot be modified (eg, smoking or alcohol consumption) would trigger negative emotions (eg, regrets) [58]. It was suggested that better quality of information would increase the likelihood of maintaining users’ engagement with an app, and consequently, they would better self-monitor their behavior [56,67]. This could be achieved by providing a wide range of information that everyone could relate to rather than facts that are already known (Q9) [72]. For example, 1 qualitative study suggested the use of health quizzes to promote engagement [75]. Health quizzes were also found promising by a large study that evaluated the uptake of a loyalty points–based health app conducted in Canada [55]. One of the intermediate objectives of that study was to improve the Canadian population’s health literacy by using health information related to quizzes. The app usage data included quiz completion rates, and the results showed that 60% of the users were highly engaged with the app by having more than 75% of health quizzes completed. Furthermore, better health literacy might enhance beliefs about consequences (eg, health outcome expectancies) [67,72] and the users’ intention to stay engaged with an app and subsequently with the behavior they target to change [72,75]. Mackert et al [53] also found that adequate heath literacy was associated with increased engagement with fitness and nutrition apps.

Users valued available statistical information [37-39,46,52,54,57,66,67,71,72,75], which was a visual or numerical summary of progress or a trend in their behavior. This included features such as step counting [71,75], the number of calories consumed [54,71], number of days spent abstaining from smoking [67], the amount of money saved by quitting smoking [72] or by reducing drinking [54], a trend in their alcohol consumption and how it changes over time [38,46,54], as well as a way to allow analysis of user data [37,75]. Being able to check their progress helped users better monitor their behavior (Q10) [37-39,71,72], and for some individuals, a positive trajectory acted as a behavioral reinforcement (Q11) [46,67]. In 2 studies, participants reported that a lack of visual representation of progress led to disengagement with the alcohol reduction app (Q12) [38,46], and 1 study on smoking cessation reported negative emotions associated with progress viewing during *a few bad days*, suggesting discouragement (Q13) [67].

#### Theoretical Domains Framework Domain: Memory, Attention, and Decision Processes

This domain focuses on the ability to retain and select information, including aspects of attention, memory, decision making, and cognitive overload (Textbox 3). Reminders [37-40,43,46,48,51,52,54,56-58,62,66,67,69-71] to engage with an app were reported to be useful for people with busy schedules and for those who tend to forget engaging with the app and, therefore, with the target behavior [37,39,43,56,67]. Individuals described being inclined to check their phones when receiving a notification [37,38,40]. Reminders positively affected behavioral regulation by prompting engagement with self-monitoring and the tracking features of the app (Q14) [37,39,40,51,54,62,67,69-71] as well as reinforcing the users by reminding them about their positive progress [40,48,51]. A microrandomized trial found that a push notification that contained a tailored health message resulted in a small increase in the engagement with a health app [43]. A large study conducted on engagement with a weight loss app found that 16% of the most engaged group used reminders, compared with 1% of the least engaged group [64]. However, not all users found reminders useful [37,39,51,56-58,66]. In the case of behaviors that are associated with stigma (eg, alcohol consumption), reminders would threaten the users’ social identity when they are received at an inappropriate time or wrong place (Q15) [38,46,54]. Therefore, the timing of when the reminders were sent as well as the language used appeared to be important conditions. If these conditions were not met, users were more likely to turn the notifications off [37,38,69] or ignore them (Q16) [56,66,67].

**Illustrative quotes (Q14-Q20) for factors mapped onto the psychological capability subcomponent of the capability, opportunity, motivation, behavior model and coded under the theoretical domains framework: memory, attention, and decision processes.**

*Engagement*

Well-designed reminders

- Quote 14: “I found it was almost like having my girlfriend there, in a good way. So you’re like, oh I haven’t done this in two days, I didn’t even realize, but my phone just reminded me. Better keep it going.” [67]
- Quote 15: “I think because they were just pinging... and I was just thinking, I don’t really want to read this right now. Obviously, and I don’t know whether they do but I guess most people check their phone when something pings in and you can be with your friends and actually maybe you wouldn’t want to be saying to your friends, I’ve just got a notification from Drinkaware.” [38]
- Quote 16: “I completely ignored them [notifications]. Actually, I’m pretty sure I had the notifications that were from the app all turned off. It just felt like a pop up, like another thing for me to click close on throughout the day. I completely paid no attention to it.” [67]

Less cognitive load

- Quote 17: “I really loved it [Couch to 5K], there was no excessive login, it was really easy you just downloaded and start you have to have your email, no password, no nothing like that, they don’t send you a bunch emails that annoy the crap out of me. Nothing.” [48]
- Quote 18: “What I’m thinking is, this better be easy, because otherwise I’m probably not going to do it. If there are too many obstacles in the way I won’t. Even though I know I need to do this, I probably won’t.” [46]

Coping games

- Quote 19: “If there was a bunch of games on the app that were there to distract you from smoking, (you could) go play 5 mins of a quick game instead of smoking.” [40]
- Quote 20: “Maybe if they had prior to like some type of like a mini game or something in there that would keep the mind occupied rather than telling you, “Don't smoke.” [72]

Regarding attention and decision processes, the findings of the studies included in the review proposed that cognitive overload should be avoided to maintain engagement with an app. An app that is less time consuming, requires minimal input, and is easy to use and log into was preferred (Q17) [37,39,46,48,50,51,54,56-58,60,66,69,71,72,75]. Additional functions that decrease the time spent on a task using an app were highly appreciated [37,39,48,50,54,56,71,72,75]. The automatization of data collection, for example, by linking apps to wearables [37] or by using the camera function to scan the barcodes to input calories [71] was found to be particularly useful in physical activity and weight management apps. An app that is easy to use and does not require extra effort would increase the intention to engage with it [39,46,48,54,56,57,74] and would improve users’ self-monitoring and self-management strategies [48,51,66,75]. Conversely, using a difficult and time-consuming app would affect the users’ perceived competence in engaging with it (Q18) [50]. Such an app often would be deleted or replaced with another app that is perceived to be easier to use [46,48,56,66,71]. Only 1 study found that users who are highly committed to change behavior (in this case, to reduce alcohol consumption) would be willing to overcome this barrier [54].

Including coping games [40,60,67,72] as distraction activities has been suggested as a helpful way to cope with cravings (smoking) [40,67,72] or with distress [60]. Some users indicated that by using their hands and minds, they expected to be preoccupied, instead of engaging with the undesirable behavior, while keeping them engaged with the app itself (Q19-Q20).

#### Theoretical Domains Framework Domain: Behavioral Regulation

Behavioral regulation refers to managing, monitoring, or changing actions or behavior (Textbox 4). Self-monitoring, the ability of an app to help monitor and regulate the target behavior [36,38-40,45,48,51,52,55,57,59,60], was found to be important in supporting behavior change. A self-monitoring feature was able to raise awareness about the number of cigarettes smoked [40,58], the amount of alcohol consumed [58], the number of steps taken [45], the mood they have [60], or users calorie intake (Q21) [48,56]. It also enhanced users’ intention to engage with an app [51,52,58], provided *self-reinforcement* [52], helped increase self-efficacy (Q22) [56,61,71], and evoked feelings of *control, security, health, empowerment, and autonomy* [54].

An established routine or regularly using an app [38,48,50,54,66] positively affected the intention to engage with an app [50] and to maintain engagement (Q23). Furthermore, safety netting [37,61,66,73], defined as the ability of an app to provide *aftercare* [66] and an option to retain an app for a potential precipitating event in the future and for relapse prevention, was found to be useful to maintain the behavior, even when the target behavior has been achieved (Q24).

**Illustrative quotes (Q21-Q24) for factors mapped onto the psychological capability subcomponent of the capability, opportunity, motivation, behavior model and coded under the theoretical domains framework: behavioral regulation.**

*Engagement*

Self-monitoring

- Quote 21: “You get a chance to see what you do on a daily basis, something you’re probably not aware of.” [56]
- Quote 22: “Because I can see I’m getting better, I use the app now, but I can see myself in the future not having to use it. Kind of like a stepping stone I guess.” [71]

Routines

- Quote 23: “Because, I’ve got a couple of other little apps that I look at on a daily, not all apps, but a little regime of four or five, you know, I check the weather and I look at my drink app, and various things like that, a little routine, so pretty much daily.” [38]

Safety netting

- Quote 24: “I think the migraine one's probably outlived its usefulness for me, but the back pain one, I could still go back to that at any time. If I started to need to monitor my pain again in a systematic way, I'd still go back to it.” [37]

### Physical Opportunity

#### Theoretical Domains Framework: Environmental Context and Resources

This domain refers to the circumstances of an individual’s situation or environment that positively or negatively affects the uptake of or engagement with health and well-being smartphone apps (Textbox 5). The availability and accessibility of a smartphone [37,40,45,49,52,57,72,78] facilitate both uptake and engagement by having a behavior change device in close proximity (Q25). Although smartphones or tablets enhance the portability and accessibility of health apps, the development of an accompanying website was suggested to reduce inequality for those who might not have the opportunity to own a smartphone (Q26) [40]. Furthermore, the results of a digital behavior change intervention study examining engagement and nonusage attrition with a physical activity program suggest that when the app was used together with the accompanying website, a higher engagement rate was observed compared with those who used the app-only or the web-only versions [49].

The low cost of an app was found to be an influential factor for uptake [37,40,47,48,56,68,72,74] so that low-income individuals would be able to afford them (Q27) [47]. In a questionnaire study in China, 1 of the top barriers to using a health app was the extra cost, having a total of 83% of patients reporting that they would not be willing to pay for a health app [68]. Nevertheless, a few participants expressed their willingness to pay a small extra fee (ie, under US $5) if, this way, they could unlock unique features otherwise not available with the free version (Q28) [37,48,56,74].

**Illustrative quotes (Q25-Q34) for factors mapped onto the physical opportunity subcomponent of the capability, opportunity, motivation, behavior model and coded under the theoretical domains framework: environmental context and resources.**

*Uptake*

Availability

- Quote 25: “It was real easy you just put it in your pocket and off you go and... you could do it at your own pace.” [45]
- Quote 26: “I feel like there would need to be a website equivalent with it (for) people who don’t have access to smartphones but do have access to public libraries. A lot of smokers are LGBTQ and a lot of LGBTQ are in poverty and homeless. The people that you want to access might not be able to access the program.” [40]

Low cost

- Quote 27: “I wouldn’t pay money for an app. I think that’s kinda stupid.” [48]
- Quote 28: “I'm prepared to pay for applications. As well as being in the software industry, I understand that it's people's livelihoods are attached to this. I use some free applications, but I often will pay for the upgraded or the purchased option.” [37]

*Engagement*

Positive tone

- Quote 29: “I had a chocolate bar today and It would say, this chocolate bar contained this much saturated fat and... I just feel really guilty now.” [71]
- Quote 30: “I think I’m more likely to listen to practical advice rather than finger wagging...” [58]
- Quote 31: “I just see it as a way to help me monitor what I’m doing and maybe give me a little kick in the pants every now again to be like, ‘By the way, that donut had ﬁve hundred calories in it. Maybe make a better choice at dinner’.” [51]

Personalization

- Quote 32: “The more I would be able to manipulate the app to be and do what I wanted or needed, for my own circumstances, the more likely I am to use it.” [59]
- Quote 33: “It must be very personalized, it's easy to find things on the Internet, but it's mostly for normal people.” [75]
- Quote 34: “Assuming that it’s customised to LGBTQ (and) it incorporates the kinds of struggles that we’ve lived through, it wouldn’t be any average quit-smoking app. The fact that it’s specific to a community... the fact that it’s LGBTQ-specific, that would help us more than if it was just a general quit-smoking app.” [40]

Numerous studies have found that interactivity and positivity of tone may be efficacious for engagement, especially when attempting to change behaviors associated with self-blame (eg, weight management) (Q29) [46,51,57-60,69,71,72]. In total, 3 studies provided evidence that an encouraging tone rather than a condescending tone was important [46,58,69]. Evidence from 1 study suggested that apps should use praise but avoid shame [51], and another study provided evidence that a relaxed tone may be beneficial and may include jokes [46]. Several studies suggested that demanding or annoying language would be ignored (Q30) [57-59], although a study of nutrition apps reported the occasional need for a tougher attitude to achieve goals (Q31) [51]. Nevertheless, careful selection of the terminology used to understand the app and what it does, such as using simple and clear language, was suggested to make a noteworthy difference in the effectiveness of the content [60,72]. Terminology around certain behaviors might make a difference. For example, it was reported that using a *nonsmoker* label as opposed to an *ex-smoker* label would increase people’s self-confidence [72]. It was suggested that unsupportive language would evoke negative emotions (eg, guilt and regret), which would affect the intention to engage with an app [46,59,71].

A personalized app was highly valued for engagement [37,38,40,47,50,52,56,57,60-62,69-72,75]. Users would want to have control over the app (Q32) [59,66,69]. They would like to be able to switch off features they do not use [37], and to use external incentives, such as uploaded photos or quotes [66,67], and to personalize their goal and how to achieve it [40]. Users would also like to choose a level where to start using a particular app. For example, a more experienced user would want to have the possibility to start a mindfulness practice at the intermediate level rather than at the beginner level [50]. Users were seeking to receive more personalized information about their current behavioral habits, demographic characteristics, long-term effects of the current behavior [38,56,60,78], and recommendations based on their tracked data [57]. Personalization can also be extended to their identity (Q33). Participants were looking for an app that is tailored to their cultural and social identities, such as LGBTQ+ people, cancer survivors, or other patients who are predisposed to have other struggles and mental health issues (Q34) [40]. Personalization to users’ needs and preferences suggested better engagement [58,59,61], whereas lack of flexibility in content was found to be a reason to stop engagement [52], and in some cases, it created frustration [71]. Furthermore, a large study found that 30% of the most frequently engaged group customized the app more, for example, uploaded pictures, than the least engaged group (2%) [63].

### Social Opportunity

#### Theoretical Domains Framework: Social Influences

Social influences are interpersonal influences (received from other individuals) that could impact an individual’s behaviors, decisions, thoughts, and feelings (Textbox 6). In 5 studies, recommendations to use an app [56-58,61,74], received from health care practitioners or trusted providers [57,61,74], friends and families [56,60,74], or by reading user reviews [56,58,74], positively affected the uptake of health and well-being apps (Q35-Q37).

Connections between an app and health practitioner support were highly valued [37,40,51,52,57,59,62,67,69,72,73]. Participants reported that counseling services should be linked to an app [40,67,69], such as an *emergency button* feature [69], whereas others have emphasized the importance of linking an app to their health care provider (Q38-Q40) [37,62]. Health practitioner support could help overcome potential barriers caused by lack of skills, such as app literacy [52]; enhance self-monitoring [52,62]; and act as reinforcement [52], having the potential to enhance intentions to engage with the app (Q40) [52,62,72].

The possibility of community networking within apps with other users or other people with similar needs has been identified in multiple studies [37,39,40,47,56,59,62,66,67,69-73,75]. It was considered an important social support by reinforcing behavior change [47,56,59,62,69,72,73] and by sharing knowledge and experiences [37,69,73,75]. This was found to increase their intention to engage with the app and, subsequently, the behavior (Q41-Q42) [62]. A large study found that the most engaged group had a mean number of 24 friends within the app, as opposed to the least engaged group (1 friend) [64]. Users’ potential social roles or group identities and personal preferences should be taken into consideration. For instance, individuals from the LGBTQ+ community [40] and cancer survivors [62] would wish to interact with people who face similar challenges (Q41). In addition, some users would not want to share information with strangers due to fear of social comparison [39,59] or social stigma [54], whereas others were more open to connecting with strangers rather than with friends or family (Q42-Q44) [56].

Evidence for the importance of embedded social media for engagement has been mixed [39,40,48,54,56,58,61,66, 67,70-72,75]. It largely depends on the individual’s attitude toward these channels and on the target behavior. Some users found this reinforcing (Q46) [40,61,71,75], whereas others did not want to engage with such features due to social stigma (eg, smoking, alcohol consumption, or weight management; Q46-Q47) [39,48,54,56,58,67,72].

Social competition [37,39,48,56,59,66,67] includes the possibility for individuals to compete with themselves (ie, their previous achievements or breaking their own records) or with other app users (Q48-Q49). A total of 5 studies suggest that the reinforcing nature of social competitions might increase the intention to engage with an app [37,48,56,59,66]. The increased engagement was anticipated when the competition is based on support by receiving encouragement from others [39,67], rather than on defeating each other, which might prompt discouragement to use the app (Q50) [67].

Several studies described that some participants felt that apps can impersonate a little person [39,45,47,48,50,56], which increased the intention to use the app (Q51-52) [45,48,50]. It was also suggested that if the app is too impersonal, it would not offer the social support the users’ need [47]. In contrast, in 2 studies, the participants were concerned about having a machine telling them what to do (Q53) [47,56].

Moreover, personal experience related to noncommunicable diseases might increase the chances of the uptake of apps. One study conducted on Latino and Asian subgroups in the United States found that the odds of downloading a health app was twice as high for those who had a family history of heart attack (odds ratio 2.02, 95% CI 1.16-3.51), compared with those who did not [41].

### Automatic Motivation

#### Theoretical Domains Framework: Reinforcement

Reinforcement is a process or action of encouraging a pattern of behavior (Textbox 7). Users reported better engagement when positive feedback was received (Q54) [37,39,45-48,51,52,54,56, 58,62,67,72]. Visual feedback of progress made users aware of their advancement in reaching their goal (Q55) [37,45,46], whereas auditory feedback was seen as encouraging during physical activity (eg, running) [37,48]. For some, instant feedback on their progress, even if it is of a positive nature, was perceived to cause pressure and potential disappointment if they were not able to reach their goal (Q56) [45,56].

Offering rewards [37,40,45,46,56-59,66,69,71,75] was found to be a useful way to increase engagement. Participants suggested including gamification elements in apps to enhance engagement [37,56,69,71,75]. Some users found intangible rewards (eg, badges) motivating (Q57) [46,56,58,59,66,71], whereas others would want to receive tangible rewards instead (eg, free t-shirt, gift cards, cash, reduction in health insurance, or vouchers provided by hospitals or doctor’s office; Q58-Q59) [40,56,58,66]. This has been partly supported by 2 quantitative studies. In 1 study, having a health insurance was associated with uptake of, but not with engagement with, health apps [42]. Another study found that when offering loyalty points, engagement increased for at least three months [55].

**Illustrative quotes (Q35-Q53) for factors mapped onto the social opportunity subcomponent of the capability, opportunity, motivation, behavior model and coded under the theoretical domains framework: social influences.**

*Uptake*

Recommendations

- Quote 35: “I’d rather ask a counselor or a doctor what they would recommend.” [61]
- Quote 36: “Most of mine [my apps] are friend recommendations, people with similar activities.” [56]
- Quote 37: “...if an app has a good rating, despite the one or two people who are not satisfied, I think it would mean that it works for the majority of people.” [58]

*Engagement*

Health practitioner support

- Quote 38: “It would help in times of crisis to be able to be in touch with a professional, or if I needed to ask health questions related to alcoholism.” [59]
- Quote 39: “I want to let others know when I’m not well, the app would help me.” [69]
- Quote 40: “The therapist helped me to find my motivation every now and then, and then I was on top of it for about a week or so, and eventually the application sort of became a part of my everyday life. Then it was pretty obvious that I would use it and then I didn't even think about whether it was hard to use it, I just did it.” [52]

Community networking

- Quote 41: “It is so important to get in touch with people who went through the same thing as you have. [...] I think that if an app for cancer survivors had a forum on it as a part of the application to motivate each other, that would be amazing.” [62]
- Quote 42: “I don't think I would share on the social media, but within the app community I think it is important to like inspire and be motivated by others.” [66]
- Quote 43: “So having some sort of platform where everyone can just say, ‘This is how I stopped’ or ‘This is how I'm trying to stop’ and then other people giving feedback saying, ‘This is good’ or, ‘This is not’.” [72]
- Quote 44: “Being able to exchange feedback with strangers with the same goal could be supportive but non-judgemental as you will probably not know the other users.” [59]

Embedded social media

- Quote 45: “Integrating it with the social media is definitely a great thing to do because they can always fall back to Facebook, Twitter, etc. And through this, people can get to share their experiences and keep an update and tell whatever experiences they may have to share. So it’s like ongoing support.” [40]
- Quote 46: “Yeah you can share on Facebook and stuff, but I hate that. I hate when apps sync to like every form of social media. I’m like really weird about social media, so, no I don’t want to share it.” [48]
- Quote 47: “Don't want to share progress on social media in case you fail.” [72]

Social competition

- Quote 48: “Whenever we do a weekend challenge, you always have a look at what the other person's doing and [their] competitive side. I just want to beat the other people I see on there, so [using the app] is quite a good motivator.” [37]
- Quote 49: “It made me want to exercise more just, as like, kinda like, a competition to see how many calories because it takes your calories off whenever you exercise so I’m like let’s see how many I can get off this time.” [48]
- Quote 50: “Someone whose successful and quit smoking isn’t any better than someone that’s struggling with it. Like, no, I didn’t-I don’t like that aspect...it just makes someone feel bad.” [67]

Impersonated app

- Quote 51: “It’s like a ‘little boss in my pocket’... that’s sort of saying ‘you know you need to get out and do this’.” [45]
- Quote 52: “It’s like your own little motivator, in a way. And it deﬁnitely, it’s like, okay it’s like a little person, but it doesn’t talk, but it’s like, you shouldn’t eat that, or it’s like you should. So I don’t know it’s, I like it—I mean, I think it’s cool. It’s like my own little motivation.” [48]
- Quote 53: “I don’t want an electronic device telling me what to do.” [56]

**Illustrative quotes (Q54-Q59) for factors mapped onto automatic motivation subcomponents of the capability, opportunity, motivation, behavior model and coded under the theoretical domains framework: reinforcement and emotions.**

*Engagement*

Feedback

- Quote 54: “I liked how it gave notifications, like every day I've got a notification saying; You're on day four of your smoking quitting history. You could do this, don’t give up. Stay loyal and stuff like that. That was quite impressive.” [72]
- Quote 55: “The big green continue at the bottom and when it moves on to the next thing I feel great, I’ve achieved something, I’ve filled something in correctly. I like that. And a nice little noise which made me think, Oh, I’m not an idiot.” [46]
- Quote 56: “The progress I didn’t make—it shows [and thus is demotivating].” [56]

Rewards

- Quote 57: “Earning badges [was] important when I was doing it...We learned as a kid, to consider [it] as [an] accomplishment.” [56]
- Quote 58: “Each time you try, you get the points. And if these points can be converted to something else. Because you know, you’re not really working for the badge but if the virtual badge can turn into something tangible, I would want that.” [57]
- Quote 59: “Well, both of them are a kind of ‘well done for doing this’, they’re both a reward, they both make you feel a bit better. But a badge, it’s a cool fact, but it’s not the same as having vouchers, where you can go and treat yourself to something you want.” [59]

#### Theoretical Domains Framework Domain: Emotions

Emotions, based on previous experiences and behavior, are a complex reaction by which people tend to respond to a personally important event or matter (Textbox 8). Curiosity [38,52,54,61] positively influences the uptake of health and well-being smartphone apps (Q60). However, in 2 studies, both targeting alcohol consumption reduction, this factor was only relevant for a specific user type: for those who were characterized as *low-risk* drinkers [38] and *noncommitters* (ie, users who did not commit to engage with the app and, thus, did not gain any benefit from it) of the app [54].

**Illustrative quote (Q60) for factors mapped onto the automatic motivation subcomponent of the capability, opportunity, motivation, behavior model and coded under the theoretical domains framework: emotion.**

*Uptake*

Curiosity

- Quote 60: “It was more like seeing an ad and just, okay I should try this — and then I found it on the internet and signed up. It was more like a fun thing. We'll see if it works. More like that.” [52]

### Reflective Motivation

#### Theoretical Domains Framework: Goals

Goals are outcomes that an individual would like to achieve to change a certain behavior (Textbox 9). Goal setting [38,39,45,48,51,54,56,58,59,66,71,74] was related to sustained engagement with health and well-being apps (Q61). Some users chose to set a goal, and mostly, this was only 1 goal at a time, so their focus would remain on 1 single aspect of change of the behavior (Q62), whereas others were more reluctant to use this feature because of fears of not being able to achieve their set goal and to avoid disappointing themselves (Q63) [38]. In general, the studies suggest that users were more determined to engage in behavior change when they had set goals [45] and believed they had successfully achieved or could achieve their goals with the help of an app by increasing their intention to use the app and by better monitoring the target behavior (Q64-Q65) [48,54,56,58,59].

**Illustrative quotes (Q61-Q65) for factors mapped onto the reflective motivation subcomponent of the capability, opportunity, motivation, behavior model and coded under the theoretical domains framework: goals.**

*Engagement*

Goal setting

- Quote 61: “I’m not good at self-discipline and exercise, so maybe this [goal setting in the app] can help me get to my goal.” [56]
- Quote 62: “I only set one goal because I was very keen to kind of remain focused on one thing. I didn’t want to come and get lost in the app using it like a game. You know, I wanted to use it for one very specific thing... I think I set it to drink probably within guidelines.” [38]
- Quote 63: “No, it didn’t appeal - probably because I thought if I put some goals in I’m probably not going to stick to it, which probably makes me sound a bit naughty.” [38]
- Quote 64: “If you set those manageable goals, so you could achieve it, if you feel like you’re actually progressing, getting something, then you’re more likely to go back.” [58]
- Quote 65: “It would encourage me to open the app on a daily basis.” [59]

#### Theoretical Domains Framework Domain: Beliefs About Consequences

This domain includes aspects related to outcome expectancies (Textbox 10). Perceived utility of the app [37,46,52,59,61,74] refers to where there is a discrepancy between what the users are looking for and what an app actually offers. It was suggested that the unmet expectations of an app would lead to disengagement and frustration with the app (Q66-Q68).

**Illustrative quotes (Q66-Q68) for factors mapped onto the reflective motivation subcomponent of the capability, opportunity, motivation, behavior model and coded under the theoretical domains framework: beliefs about consequences.**

*Engagement*

Perceived utility of the app

- Quote 66: “I do have some apps I don't use often, mainly because they've kind of bored me in a way. I'll just do an example: one fitness app shows you how to lose weight, but the way it's describing it, it's not what I'm after. It's one of those free apps I bought that—I thought [the fitness app] would be great, but when you actually use it, it's not the same.” [37]
- Quote 67: “I think that’s where it let itself down for me. Once I’d played with it, once I tried the game, done the identity and whatnot, there wasn’t much else there for me.” [46]
- Quote 68: “It [mindfulness app] didn’t add anything...I guess it didn’t detract, it didn’t make anything worse, but it didn’t add anything to my armoury, I guess, my tool kit, as keeping myself sane, I suppose, it didn't add.” [61]

### Other Factors

There were a number of sociodemographic factors that did not fit clearly under the components of the COM-B model.

#### Sociodemographic Factors

Apps were more frequently downloaded by women than men, with the percentage ranging from 59% to 74% [38,41,49,53,55,63], although 1 study found that being male was associated with using an app to manage alcohol consumption [65]. Being younger than 44 years was associated with a higher level of uptake and engagement [38,41,42,44,49,53,55,63,64] than older adults. Living in an urban area [42,44,55]; having a better education level, such as having high school education or higher [41,42,44,64] and college degree or higher [41,53]; and having a higher income [44] were also associated with better engagement with health and well-being apps.

## Discussion

### Principal Findings

This is the first systematic review to conduct a theoretical analysis using the COM-B model of factors influencing the uptake of and engagement with health and well-being apps. The findings from this review suggest that there are 26 key factors across the constructs of capability, opportunity, and motivation that influence the uptake of and engagement with these types of apps, which were found to be important for a wide range of populations and behaviors.

Our review replicates previous findings in the wider literature on digital behavior change interventions. The core findings of our review suggest that attention should be perhaps shifted mainly to the support and guidance offered to new and existing users of health and well-being apps. We found that support and guidance of uptake can be targeted by increasing their awareness of health apps through, for example, recommendations received from health practitioners. In line with the findings of previous reviews, help with initial engagement could be achieved by improving the users’ app literacy skills and by providing knowledge [14,17]. We present knowledge in a novel way by breaking it down to instructions on how to use it (ie, user guidance), advice related to the target behavior or condition (ie, health information), and information on their progress or data (ie, statistical information). This suggests that allowing access to users to different information that serves different purposes (eg, health benefits vs progress data) would enhance their engagement through different channels, such as guidance, support, and education.

Potentially, one of the most important factors for engagement identified in this review is health practitioner support. In line with the emerging evidence from the human-computer interaction (HCI) literature, we found that an app coupled with human support [14,17] was likely to be more effective by increasing the intervention effectiveness and engagement [78,79]. Alternatively, human support can be impersonated by embedded artificial intelligence (AI) features. A recent experimental study found that a supportive AI-powered chatbot doubled the engagement with a smoking cessation app and increased its effectiveness [80]. This suggests that embedded human support or features that mimic human support might lead to greater engagement with digital behavior change tools.

Behavior change techniques, widely reported by others previously [14,17-19], were also identified as important factors to sustain engagement, including self-monitoring, feedback, goal setting, reminders, rewards, and social support. However, we found that not all of these have a positive effect. Reminders and social support factors (embedded social media and social competition) are not universally useful and might cause disengagement or even harm by triggering negative emotions. One plausible explanation is that the participants of the studies included may or may not have real-life experience with health and well-being apps. Some of the included studies examined participants’ perceptions about a hypothetical app or an app that was planned to be developed. These studies relied on the participants’ opinion of what they think would be important for them in terms of uptake of and engagement with health and well-being apps, rather than sharing their lived experiences with such tools. For example, reminders were found useful in all the studies targeting a hypothetical app, as opposed to those that were researching engagement with an app that had been used by the participants, where opinions about reminders were mixed, with some users finding them annoying. Another explanation is that the importance of these factors might be dependent on the target behavior. For example, people using apps that target mental health might not want to engage with social competition features or to share their progress or experiences on social media. This suggests that some of the identified factors in this review might be behavior dependent.

Another interesting finding, not identified in previous literature, is the safety netting characteristic of an app. This characteristic could promote long-term engagement rather than short goal-oriented engagement. The user could disengage at any time and reengage at a later stage when needed. This feature might be particularly useful for addiction research targeting relapse prevention strategies.

No factors were coded directly under 4 out of the 14 TDF domains (optimism, social identity, beliefs about capabilities, and intentions). However, 2 of these were highlighted in this review. We described how several factors coded under different domains affect intentions (eg, having adequate app literacy skills or user guidance provided to the user), in a manner similar to how emotions, other than curiosity, affect engagement with an app (eg, lack of app literacy skills triggers negative emotions, some found reminders annoying, or some fear of social comparison related to sharing on social media). We also found that aspects of the factor *personalization to needs* also include social identity aspects. Some communities (LGBTQ+ and cancer patients) prefer an app that is personalized to their social identity. Although social identity, in this case, was judged to be a weak factor to list it independently. In terms of the other two absent domains, factors under beliefs in their capabilities and optimism might be less relevant for uptake and engagement with health apps, or the studies may have missed them out, or, potentially, we failed to identify them from the included studies.

The importance of promoting equality and embracing cultural diversity has been partially identified previously [18]. Several studies in this review reported that apps should be provided at a low cost to users. It was suggested that multiculturalism should be embraced, and regional languages should be added. The concern of inequality for those who do not own a smartphone was also raised in this review [40]. An accompanying website was suggested as an alternative for homeless people who would not have access to a smartphone but may have access to the internet through nonprofit organizations, charities, or community libraries.

### Strengths and Limitations

One major strength of this paper is that it adhered to the best practice processes for undertaking reviews by following the PRISMA guidance and Cochrane handbook [27,29]. By including all study designs, we were able to pool together and triangulate evidence and provide a novel and powerful synthesis of different study designs.

The use of theoretical frameworks is another strength. Other theoretical models were considered for this review, including the technology acceptance model [81] and the HCI models and theories [82]. However, the COM-B and TDF present advantages owing to their dynamic nature and by explaining the influences between components as they were developed from, and to represent, all theoretical components in behavior change–related models and theories. COM-B was explicitly developed to inform behavior change interventions through its connection to the Behavior Change Wheel [83], a tool that provides guidance on designing behavior change interventions. The factors identified under the components of the COM-B model allow easy identification of the intervention functions to target increased uptake of and engagement with health and well-being smartphone apps.

This review has several limitations. The review focused on 4 major behaviors related to prevention (smoking, alcohol consumption, physical activity, and diet) and mental health and well-being and could not capture other prevention type behaviors (eg, fall prevention). Factors relating to the uptake and engagement of apps focusing on other behaviors or conditions may differ from those found in this review and warrant further investigation.

Although we captured a wide range of populations, most of the included studies were carried out in high-income countries. Therefore, the findings might not be transferable to low- and middle-income countries or to other cultures. The quality of the studies was mixed. In some qualitative studies, the authors provided interpretations of their findings without an explicit quotation to support them. These interpretations were handled with care and were often ignored when no further explanation was provided about a concept. This might have led to losing some potentially important factors, not identified otherwise.

### Policy and Practice: Recommendations and Implications

The findings of this review can inform app developers and researchers on how to develop health and well-being smartphone apps to better support behavior change and manage and monitor different physical and mental health conditions in adults.

This review may also have implications for policies that target prevention using digital technologies. Apps are an easy way to provide health-promoting behaviors and may play an important role in prevention strategies. For example, the UK government has recently published a Green Paper entitled *Advancing our health: prevention in the 2020s*, which shifted their focus from *cure to prevention,* committing to encourage the population to live a healthier life [84]. Additionally, the *Long Term Plan* policy document of the NHS in the United Kingdom dedicates an entire chapter to prevention programs and includes plans on digitally delivered methods to improve access to information, education, and intervention [85].

As part of prevention and health management strategies, the NHS and partners have created a pool of health and well-being apps for the individuals to access (NHS Apps Library). This research could help people access effective apps that people will remain engaged with, although the extent to which the population is open to use these portals for uptake is yet unknown and something worth investigating in the future.

A number of important themes are described in the projects and policy documents mentioned above. Some relate to digital health, for example, with an aim to reduce health inequalities [84] or to improve population health with personalized content and tailored lifestyle advice [85]. Our review suggests that app literacy skills are important for uptake. Enhancing app literacy skills for the elderly (eg, drop-in sessions in community settings) might be a feasible way to reduce health inequalities. Furthermore, some of the engagement-related factors might suggest the use of tailored lifestyle advice to address health behaviors, for example, by receiving personalized content within the app and web-based or offline help or advice from health practitioners as well as receiving recommendations for use of health apps from their health care professionals and general practitioner practices.

Therefore, our findings could inform stakeholders in public health, policy makers, and providers of health and well-being smartphone app portals to provide additional support for the uptake of and engagement with these digital interventions for adults.

Recommendations for stakeholders in public health, policy makers, and health and well-being app developers derived from the findings of this review can be found in Table 3.

**Table 3:** Recommendations for stakeholders in public health, policy, industry, health care, and health and well-being app development.

Component	Policy makers/industry/health care providers might want to consider	App developers might want to consider
Capability	Improving app literacy skills Increasing awareness of effective health and well-being apps, by advertising offline (eg, general practitioner practices) and web-based (eg, social media)	Promoting less cognitive load by enabling automatization of data collection Including user guidance that can be deactivated once the functionality of the app has been achieved (eg, help button) Including content that targets education, health prevention, and health consequences related to the behavior that is targeted to change Including statistical information (eg, graphs, percentages, and numbers) about the user’s progress Including well-designed reminders where the user can choose the time and frequency of receiving it Including the self-monitoring feature that enables users to create routines Including a *safety netting* feature that allows users to fall back on, even when the target behavior has been achieved
Opportunity	Providing web-based or offline health practitioner support Providing recommendations for health and well-being apps by health care professionals Offering apps for free or at a low cost	Allowing the provision of health professional support within the app Allowing community networking within the app with other users Organizing competition and challenges for users to opt in to Avoiding automatic synching with the embedded social media (when applicable) Personification of the app, by designing human-type attributes Offering apps for free or at a low cost Offering personalization of the app according to their demographics and individual and cultural needs
Motivation	Offering tangible rewards, such as points that could be used as a discount in pharmacies or at other health- and well-being–related domains or health insurance providers Providing a meaningful title and clear description of what the app does and what can offer, and how can help the user	Providing positive, nonjudgmental, constructive, and informative feedback Include gamification elements and offering rewards Including goal-setting features (when applicable) Providing a meaningful title and clear description of what the app does and what can offer, and how can help the user

### Future Research

Although some of the factors identified and presented in the Results section appear to have a positive influence on uptake and engagement, there are mixed findings that might benefit from further investigation, such as reminders, embedded social media, and social competition. In the studies included in the review, descriptions of notification-type messages, such as reminders, feedback, push notifications, and other notifications, were used interchangeably, and it was not always clear which notifications were being referred to. Consistent terminology would help eliminate doubt around these concepts in the future. Issues around equality and diversity were highlighted in a few studies as something future research should address. Further work is also needed to aid our understanding of how to avoid digital health widening inequalities through the exclusion of individuals who face a financial barrier to owning a smartphone or to purchasing an app, or who do not possess the skills to use one.

### Conclusions

This is the first systematic review to investigate factors that influence the uptake of and engagement with health and well-being smartphone apps. We identified 26 factors that are relevant to a wide range of populations and different behaviors. These have clear implications for improving population health and targeting health inequalities. We provide a list of recommendations built on the identified factors to guide app developers, health app portal developers, and policy makers when commissioning, developing, and optimizing health and well-being smartphone apps. These can help address the issues of suboptimal uptake and engagement, which currently constrain the public health benefit of apps.

## Appendix

Multimedia Appendix 1 A visual representation of mapping the capability, opportunity, motivation, behavior model onto the Theoretical Domains Framework.

Multimedia Appendix 2 Preferred Reporting Items for Systematic Reviews and Meta-Analyses checklist.

Multimedia Appendix 3 Medical Literature Analysis and Retrieval System Online, or MEDLARS Online search strategy.

Multimedia Appendix 4 Characteristics of the studies included in the review.

Multimedia Appendix 5 Quality assessment of the studies included in the review.

## Abbreviations

COM-B model: capability, opportunity, motivation, behavior model
TDF: theoretical domains framework
HCI: human-computer Interaction
PRISMA: Preferred Reporting Items for Systematic Reviews and Meta-Analyses
MMAT: mixed methods appraisal tool
LGBTQ+: lesbian, gay, bisexual, transgender, queer, and other spectrum of sexuality and gender
MEDLINE: Medical Literature Analysis and Retrieval System Online, or MEDLARS Online
NHS: National Health Service
PHE: Public Health England
