# Supplementary material for: Influences on the Uptake of and Engagement With Health and Well-Being Smartphone Apps: Systematic Review
Source: J Med Internet Res. 2020 May 29;22(5):e17572. doi: 10.2196/17572 (PMC7293059; doi:10.2196/17572)

Multimedia Appendix 1. A visual representation of mapping of the COM-B model onto the Theoretical Domains Framework.

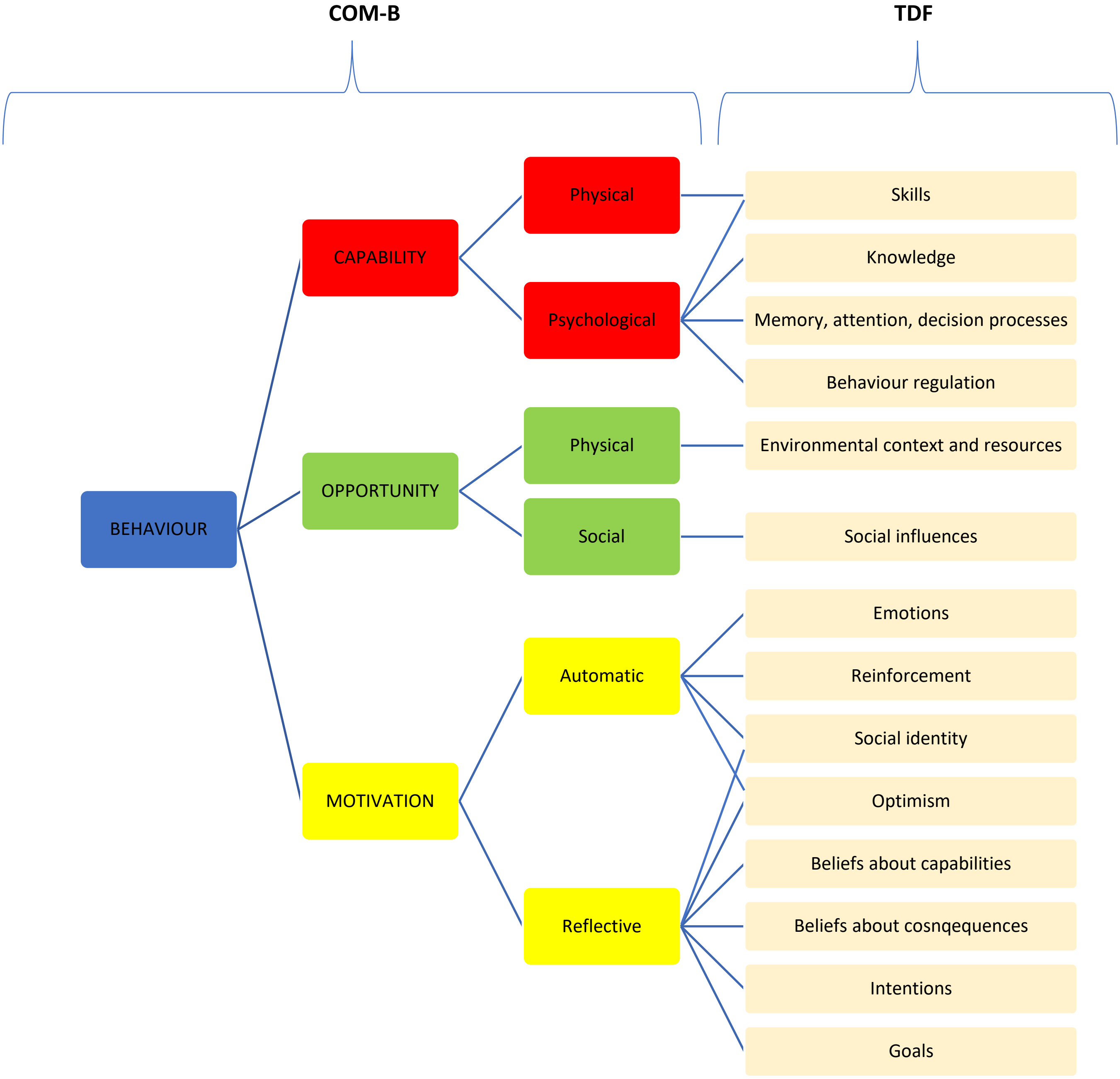

Supplement: Multimedia Appendix 1 [file jmir_v22i5e17572_app1.pdf]
