# Supplementary material for: Influences on the Uptake of and Engagement With Health and Well-Being Smartphone Apps: Systematic Review
Source: J Med Internet Res. 2020 May 29;22(5):e17572. doi: 10.2196/17572 (PMC7293059; doi:10.2196/17572)
Supplement: Multimedia Appendix 3 [file jmir_v22i5e17572_app3.pdf]

Multimedia Appendix 3. Search strategy applied in MEDLINE:

1. MeSH: health promotion, health behaviour, health education (/explode), smoking cessation OR

((Behaviour adj2 change) OR (Behavior adj2 change) OR (behaviour adj2 change adj2 technique*) OR (behavior adj2 change adj2 technique*) OR (behaviour change strategy*) OR (behavior change strateg*) OR health behaviour OR health behavior OR health education OR health promotion OR health prevent* OR BCT* OR behaviour* intervention* OR behaviour* modification* OR (health adj2 campaign*) OR diet* OR nutrition* OR (healthy adj2 eating*) OR exercise* OR (physical adj2 activit*) OR (physical adj2 inactivit*) OR (alcohol adj2 misuse) OR drink* OR (smok* adj2 cessation) OR (stop adj2 smok*)OR tobacco* OR mood OR depress* OR anxi* OR wellbeing).ti,ab,kw.

2. MeSH: Mobile application (/explode) OR

(Smartphone* OR (mobile adj phone) (Smartphone* adj2 app*) OR (mobile adj2 app*) OR mhealth OR (mobile adj2 technolog*) OR (mobile adj2 tablet*) OR (mobile adj2 health*)).ti,ab,kw.

3. (uptake* OR engage* OR use* OR adher* OR enrol* OR participat* OR commitment OR connect* OR download* OR disconnect* OR discontinue* OR abandon* OR disrupt* OR interrupt* OR quit* OR terminate OR disengage* OR detach* OR withdraw* OR usage* OR pageview* OR screenview* OR login* OR log-in*).ti,ab,kw.
4. 1 AND 2 AND 3
