# Supplementary material for: Influences on the Uptake of and Engagement With Health and Well-Being Smartphone Apps: Systematic Review
Source: J Med Internet Res. 2020 May 29;22(5):e17572. doi: 10.2196/17572 (PMC7293059; doi:10.2196/17572)
Supplement: Multimedia Appendix 4 [file jmir_v22i5e17572_app4.pdf]

Multimedia Appendix 4. Characteristics of the studies included in the review: *Influences on the Uptake of and Engagement with Health and Well-being Smartphone Apps: A systematic Review*

Studies	Location	Study aim	App used (name if applicable)	Participants	Methods or design and analytic approach
Anderson et al 2016 [37]	Australia	To explore experiences of health app users	Nonspecific health apps	Adults in the general population; N=22; female n=15; age groups: 18-25 years, n=4; 26-35 years, n=13; 46-55 years, n=2; and ≥55 years, n=1	Semistructured interviews; thematic analysis
Attwood et al 2017 [38]	United Kingdom	To examine patterns of app usage over time and to explore app users' views of the app	Alcohol reduction (Drinkaware)	Exsiting app users; N=119,713, (interview participants N =21); female (%): 59.3, (interview part: 12); age groups: 31%, 35-44 years	Mixed methods approach (secondary data analysis of Drinkaware database and semistructured interviews); ANOVA[a], regression, *t* test, framework analysis
Baretta et al. 2019 [39]	Italy	To examine users' need and preferences regarding their engagement with physical activity apps	Physical activity (Runtastik, Edumondo, Runkeeper)	Adults in the general population; N=20; female (%): 45; mean age 39.8 years (SD 7)	Longitudinal, single-arm design with think-aloud methodology and interview techniques; thematic analysis
Baskerville et al 2016 [40]	Canada	To explore LGBTQ+[b] communities' perception of a smoking cessation app	Smoking cessation	LGBTQ+ youth and adults; N focus groups=204; female (%): 39, male (%) 26.6, trans female (%): 3.7, trans male (%): 6.9, two spirit (%): 4.1, queer (%): 14.7, 0.5% intersex (%): 0.5, 4.6% other (%): 4.6; age groups: 8.8%, 16-18 years; 91.2%, 18-29 years	Focus groups (n=24); framework analysis
Bender et al 2014 [41]	United States	To examine factors predicting uptake with health apps among ethnic minorities	Nonspecific health apps	Ethnic minorities in the United States (Caucasians, Latinos, and Koreans); N=904; female (%): 64.3; mean age 44 years (SD 16.1)	Cross-sectional survey; descriptives, regression
Bhuyan et al 2016 [42]	United States	To explore the use of mHealth[c] apps for heath seeking behavior among US adults	Nonspecific health apps	Adults in the general population; N=3677; female (%): 51.7; age groups: 30.8%, under 35 years; 17.2%, 35-44 years;	Secondary data analysis of a nationally representative sample (Health Information National Trends Survey—cycle 4); descriptives, regression

				18.9%, 45-54 years; 15.8%, 55-64 years; 17.4 >65 years	
Bidargaddi et al 2018 [43]	United States	To assess the effectiveness of push notifications on engagement	Well-being app (JOOL)	Existing app users; N=1255; female (%): 63.97; age groups: 28.86%, < 30 years; 42.44%, 30-50 years; 28.70%, > 50 years	Microrandomized trial; regression
Carroll et al 2017 [44]	United States	To describe sociodemographic characteristics with health app use, predictors of health app use	Nonspecific health apps	Adults in the general population; N=3519; female (%): 51.62; age groups: 65.62%, 18-44 years; 34.38%, > 45 years	Secondary data analysis of a nationally representative sample (Health Information National Trends Survey—cycle 4); regression
Casey et al 2014 [45]	Ireland	To explore patients views of using a smartphone app to promote physical activity in primary care	Physical activity (SMART MOVE)	Adult patients in primary care; N=1255; female (%): 75%; mean age 42 years (age range 17-62)	Semistructured interviews; framework analysis
Crane et al 2017 [46]	United Kingdom	To understand the usability of the app	Alcohol reduction (Drink Less)	Adult excessive drinkers and users of the Drink Less app; N=24; female (%): 50; mean age (think-aloud) 42 years; mean age (interviews) 40 years	Think-aloud and semistructured interviews; thematic analysis
Gorton et al 2011 [47]	New Zealand	To explore a potential weight loss management intervention on smartphone	Weight management	Adults in the general population; N=306 (focus groups N=54); % female (% survey): 77 (% focus group: 76); age groups (survey): 20%, 16-30 years; 51%, 31-50 years; 28%, ≥51 years; age groups (focus group): 35%, 16-30 years; 50%, 31-50 years; 15%, ≥51 years	Mixed methods approach (cross-sectional survey and focus groups [n=10]); descriptives, thematic analysis
Gowin et al 2015 [48]	United States	To describe the use of health apps among students	Weight management and physical activity	College students; N=27; female (%): 78; age groups: 70%, 18-20 years; 22%, 21-23 years; 8%, 24-26 years	Semistructured interviews; grounded theory

Guertler et al 2015 [49]	Australia	To examine the engagement with physical activity promotion app and identify sociodemographic factors of nonengagement	Physical activity (10,000 steps)	App users, N=1451; female (%): 7 2.43; mean age 38.3 years (SD 11.1)	Secondary data analysis of the *10,000 Steps* database; ANOVA, chi-square, regression
Laurie and Blandford 2016 [50]	United Kingdom	To understand users' experiences with mindfulness app	Mindfulness (Headspace)	Adults in the general population; N=16; % female (%): 68.75; mean age, 32.5 years (age range 25-38)	Semistructured interviews; grounded theory
Lieffers et al 2018 [51]	Canada	To understand the experiences of adults who have used a nutrition app previously	Weight management	Adults in the general population; N=24; % female (%), 79; age groups: 63%, 18-30 years; 25%, 31-50 years; 13%, 51-70 years	Semistructured interviews; content analysis
Ly et al 2014 [52]	Sweden	To explore participants' views of a mental health app	Depression	Adults with major depression; N=12; female (%): 50; mean age 37.9 years (age range 21-59)	In-depth interviews; thematic analysis
Mackert et al 2016 [53]	United States	To determine the association between health literacy and app engagement	Fitness and weight management	Adults in the general population; N=4974; female (%): 57.74; mean age 43.5 years (SD=16.7)	Cross-sectional survey; cross-tabulation analysis, regression
Milward et al 2018 [54]	United Kingdom	To understand why and how participants engaged with the app, to understand facilitators and barriers to engagement with the app, to understand how the app impacted drinking behavior, and to identify typologies of users (engagement)	Alcohol reduction (BRANCH)	Participants of a randomized controlled trial; N=20, female (%): 80; mean age 24 years (SD=3)	Semistructured interviews; framework analysis

Mitchell et al 2017 [55]	Canada	To evaluate uptake with a loyalty points–based health app and to describe sociodemographic characteristics of the users	Multipurpose health app (Carrot Rewards)	App users; N=57,885; % female, 62.96%; age groups: 2.4%, 13-17 years; 20.65%, 18-24 years; 33.69%, 25-34 years; 20.11%, 35-44 years; 13.17%, 45-54 years; 7.22%, 55-64 years; 2.74% >65 years	Process evaluation; descriptives
Peng et al 2016a [56]	United States	To better understand a more diverse pool of users' perception of health apps	Nonspecific health apps	Adults in the general population; N=44; female (%): 65; mean age 37.2 years (SD 15.7)	Focus groups (n=6) and interviews (n=5); thematic analysis
Peng et al 2016b [57]	United States	To explore the perception of rural adults with diabetes regarding apps to manage their condition	Nonspecific health apps	Adults with diabetes; N=18; female (%): 72.2; mean age 54 years (SD 12.7)	Focus groups (n=4); thematic analysis
Perski et al 2017 [58]	United Kingdom	To explore participants' choices of health apps and to identify important features of engagement	Smoking cessation and alcohol reduction	Adults in the general population; N=20; % female (%): 60; mean age (SD), 29.7 (SD 9.2) years	Think-aloud and semistructured interviews; thematic analysis
Perski et al 2018 [59]	United Kingdom	To explore the more important features of engagement	Alcohol reduction	Adults in the general population; N=132 (focus group: n=9); female (%): 49.2 (focus group %: 77.8); age groups (survey): 10.6%, 18-24 years; 24.2%, 25-34 years; 34.1%, 35-44 years; 21.2%, 45-54 years; 6.8%, 55-64 years; 3%, ≥65 years; age groups (focus group): 44.4%, 18-24 years; 33.3%, 25-34 years; 22.2%, 45-54 years	Mixed methods approach (Web-based survey and focus groups, n=3); interclass correlation coefficient, thematic analysis
Peters et al 2018 [60]	Australia	To explore participants' preferences of a mental health app	Well-being	Adult workers of male-dominated industry; N=60; female (%): 8%; Mean age 47 years (age range 26-65)	Participatory study: workshops (n=6); thematic analysis

Pung et al 2018 [61]	Australia	To explore mobile app use among patients with depressive symptoms	Depression	Patients of primary care presenting depressive symptoms; N=16; % female (%): 58; age groups: 19%, <25 years; 44%, 25-44 years; 38%, 45-65 years	Semistructured interviews; thematic analysis
Puszkiewitz et al 2016 [62]	United Kingdom	To assess cancer survivors' attitudes toward a physical activity app, to understand how the app could be adapted to their needs, to understand how to increase their physical activity level using the app	Physical activity	Adult cancer survivors; N=11; female (%): 89; mean age 45 years (SD=9.4)	Mixed methods approach (1-arm pre-post design and semistructured interviews); Wilcoxon sign rank test; thematic analysis
Serrano et al 2017 [63]	United States	To explore features of the app that influence engagement and to describe the characteristics of the users	Weight loss app (Lose it!)	App users; N=1,011,008	Secondary data analysis of a cross-sectional data; Classification and Regresion Tree analysis, descriptives, regression
Sharpe et al 2018 [64]	United States	To determine factors associated with uptake of an alcohol reduction app among persons living with HIV	Alcohol reduction	Adult population living with HIV; N=757; female (%): 35; age groups: 18%, 18-34 years; 20%, 35-44 years; 41%, 45-54 years; 21%, ≥55 years	Secondary data analysis of a cross-sectional survey data of a longitudinal cohort study (Florida cohort study); descriptives, regression
Smahel et al 2017 [65]	Czech Republic	To reveal characteristics regarding use of heath apps	Fitness and weight management	Adults of the general population; N=406; female (%): 86.9; mean age 23.8 years (SD=5.3)	Cross-sectional survey; descriptives, regression
Solbrig et al 2016 [66]	United Kingdom	To explore experiences and wishes regarding weight management using apps	Weight management (FIT)	Adults of the general population; N=24; female (%): 79.2; mean age 30 years (age range 19-70)	Focus groups (n=6); thematic analysis
Struik et al 2018 [67]	Canada	To understand the interaction and experiences with the app	Smoking cessation (Crush the Crave)	App users; N=31; female (%): 42; mean age 24 years (SD=2.72)	Semistructured interviews; framework analysis

Sun et al 2017 [68]	China	To investigate the current usage, willingness to use, and barriers to use a physical activity app	Physical activity	Adult patients with chronic disease; N=218; female (%): 61; mean age 44.6 years (age range 20-69)	Cross-sectional survey; descriptives, chi-square
Switsers et al 2018 [69]	Belgium	To examine the needs of adults with bipolar disorder regarding apps	Mental health	Adults with bipolar disorder; N=16; female (%): 56.3; mean age 41.8 years (age range 21-69)	Focus groups (n=7); thematic analysis
Taki et al 2019 [70]	Australia	To examine how app characteristics influence engagement	Weight management (GH[d])	Female app users; N=18, mean age 30.9 years (age range 21-38)	Semistructured interviews; thematic analysis
Tang et al 2015 [71]	United Kingdom	To explore young adults' experiences of using apps	Weight management	Adults of the general population; N=19; female (%): 47.37; age range 19-33 years	Semistructured interviews; thematic analysis
Tudor-Sfetea et al 2018 [72]	United Kingdom	To explore individuals' perceptions of different smoking cessation apps	Smoking cessation (Quit Genius and NHS[e] Smokefree)	App users; N=15 (Quit Genius) and N=14 (NHS Smokefree); female (%): 13.3 (Quit Genius) and 14.3 (NHS Smokefree); mean age 25.07 years (Quit Genius) and 24.21 years (Quit Genius)	Semistructured interviews; thematic analysis
Wang et al 2018 [73]	China	To explore app engagement and to understand people's views about app containing health information	Pregnancy health apps	Pregnant women from secondary care; focus groups N=28, mean age 29.6 years (SD=3.1); survey N=535, mean age 30.6 years (SD=3.6)	Survey and focus groups (n=4); descriptives, logistic regression, thematic analysis
Webcredible Report, 2016 (unknown authors) [74]	United Kingdom	To understand why people use health apps, how they choose them, what factors influences their choice and engagement	Nonspecific health apps	Adults in the general population; N=300 (focus group: n=12); female (%): 42; age range 33-60 years	Mixed methods approach. (Web-based survey and focus groups [n=2]); analysis used unreported
Woldaregay et al [75]	Norway	To explore motivational factors of user engagement with health apps	Nonspecific health apps	Adults of the general population; N=16; female (%): 50; Age range 21-55 years	Semistructured interviews; thematic analysis

Xie et al 2018 [76]	China	To examine the prevalence, extent, and demographics of health app use	Nonspecific health apps	Adults of the general population; N=633; female (%): 48.5; age groups: 24.6%, 18-29 years; 25%, 30-44 years; 24.6%, 45-59 years; 25%, ≥60 years	Cross-sectional survey; descriptives, regression
Zeng et al 2015 [77]	United States	To examine demographical, psychological, and behavioral predictors of the use of app	Smoking cessation (SmartQuit)	App users; N=98; female (%): 53; mean age 41.5 years (SD=12)	Secondary data analysis of the SmartQuit trial's data (intervention arm); descriptives, regression

[a]ANOVA: analysis of variance.

[b]LGBTQ+: lesbian, gay, bisexual, transgender, queer, and other spectrum of sexuality and gender.

[c]mHealth: mobile health.

[d]GH: Growing Healthy.

[e]NHS: National Health Service.

References

38. Attwood S, Parke H, Larsen J, Morton KL. Using a mobile health application to reduce alcohol consumption: a mixed-methods evaluation of the drinkaware track & calculate units application. BMC Public Health 2017;17(1):394. PMID: 28511698

39. Baretta D, Perski O, Steca P. Exploring Users' Experiences of the Uptake and Adoption of Physical Activity Apps: Longitudinal Qualitative Study. JMIR Mhealth Uhealth 2019;7(2):e11636 doi: 10.2196/11636. PMID: 30735143

40. Baskerville NB, Dash D, Wong K, Shuh A, Abramowicz A. Perceptions Toward a Smoking Cessation App Targeting LGBTQ+ Youth and Young Adults: A Qualitative Framework Analysis of Focus Groups. JMIR Public Health and Surveillance 2016;2(2):e165. PMID: 27864164

41. Bender MS, Choi J, Arai S, Paul SM, Gonzalez P, Fukuoka Y. Digital technology ownership, usage, and factors predicting downloading health apps among caucasian, filipino, korean, and latino americans: the digital link to health survey. JMIR MHealth and UHealth 2014;2(4):e43. PMID: 25339246

42. Bhuyan SS, Lu N, Chandak A, et al. Use of Mobile Health Applications for Health-Seeking Behavior Among US Adults. Journal of Medical Systems 2016;40(6):153. PMID: 27147516

43. Bidargaddi N, Almirall D, Murphy S, et al. To Prompt or Not to Prompt? A Microrandomized Trial of Time-Varying Push Notifications to Increase Proximal Engagement With a Mobile Health App. JMIR Mhealth Uhealth 2018;6(11):e10123 doi: 10.2196/10123. PMID: 30497999

44. Carroll JK, Moorhead A, Bond R, LeBlanc WG, Petrella RJ, Fiscella K. Who Uses Mobile Phone Health Apps and Does Use Matter? A Secondary Data Analytics Approach. Journal of medical Internet research 2017;19(4):e125. PMID: 28428170

45. Casey M, Hayes PS, Glynn F, et al. Patients' experiences of using a smartphone application to increase physical activity: the SMART MOVE qualitative study in primary care. British Journal of General Practice 2014;64(625):e500-8. PMID: 25071063

46. Crane D, Garnett C, Brown J, West R, Michie S. Factors Influencing Usability of a Smartphone App to Reduce Excessive Alcohol Consumption: Think Aloud and Interview Studies. Frontiers in Public Health 2017;5:39. PMID: 28421175

47. Gorton D, Dixon R, Maddison R, Mhurchu CN, Jull A. Consumer views on the potential use of mobile phones for the delivery of weight-loss interventions. J Hum Nutr Diet 2011;24(6):616-9 doi: 10.1111/j.1365-277X.2011.01163.x. PMID: 21781188
48. Gowin M, Cheney M, Gwin S, Franklin Wann T. Health and Fitness App Use in College Students: A Qualitative Study. American Journal of Health Education 2015;46(4):223-30 doi: 10.1080/19325037.2015.1044140. PMID:
49. Guertler D, Vandelanotte C, Kirwan M, Duncan MJ. Engagement and Nonusage Attrition With a Free Physical Activity Promotion Program: The Case of 10,000 Steps Australia. Journal of medical Internet research 2015;17(7):e176. PMID: 26180040
50. Laurie J, Blandford A. Making time for mindfulness. International Journal of Medical Informatics 2016;96:38-50.
51. Lieffers JRL, Arocha JF, Grindrod K, Hanning RM. Experiences and Perceptions of Adults Accessing Publicly Available Nutrition Behavior-Change Mobile Apps for Weight Management. Journal of the Academy of Nutrition & Dietetics 2018;118(2):229-39.e3. PMID: 28625662
52. Ly KH, Janni E, Wrede R, et al. Experiences of a guided smartphone-based behavioral activation therapy for depression: A qualitative study. Internet Interventions 2015;2(1):60-68.
53. Mackert M, Mabry-Flynn A, Champlin S, Donovan EE, Pounders K. Health Literacy and Health Information Technology Adoption: The Potential for a New Digital Divide. Journal of medical Internet research 2016;18(10):e264. PMID: 27702738
54. Milward J, Deluca P, Drummond C, Kimergård A. Developing Typologies of User Engagement With the BRANCH Alcohol-Harm Reduction Smartphone App: Qualitative Study. JMIR Mhealth Uhealth 2018;6(12):e11692 doi: 10.2196/11692. PMID: 30545806
55. Mitchell M, White L, Oh P, et al. Uptake of an Incentive-Based mHealth App: Process Evaluation of the Carrot Rewards App. JMIR MHealth and UHealth 2017;5(5):e70. PMID: 28559224
56. Peng W, Kanthawala S, Yuan S, Hussain SA. A qualitative study of user perceptions of mobile health apps. BMC public health 2016;16(1):1158. PMID: 27842533
57. Peng W, Yuan S, Holtz BE. Exploring the Challenges and Opportunities of Health Mobile Apps for Individuals with Type 2 Diabetes Living in Rural Communities. Telemedicine journal and e-health : the official journal of the American Telemedicine Association 2016;22(9):733-38. PMID: 26982017
58. Perski O, Blandford A, Ubhi HK, West R, Michie S. Smokers' and drinkers' choice of smartphone applications and expectations of engagement: a think aloud and interview study. BMC medical informatics and decision making 2017;17(1):25. PMID: 28241759
59. Perski O, Baretta D, Blandford A, West R, Michie S. Engagement features judged by excessive drinkers as most important to include in smartphone applications for alcohol reduction: A mixed-methods study. DIGITAL HEALTH 2018;4:2055207618785841 doi: 10.1177/2055207618785841. PMID: 31463077
60. Peters D, Deady M, Glozier N, Harvey S, Calvo RA. Worker Preferences for a Mental Health App Within Male-Dominated Industries: Participatory Study. JMIR Mental Health 2018;5(2):e30. PMID: 29695371
61. Pung A, Fletcher SL, Gunn JM. Mobile App Use by Primary Care Patients to Manage Their Depressive Symptoms: Qualitative Study. Journal of Medical Internet Research 2018;20(9):e10035. PMID: 30262449
62. Puszkiewicz P, Roberts AL, Smith L, Wardle J, Fisher A. Assessment of Cancer Survivors' Experiences of Using a Publicly Available Physical Activity Mobile Application. JMIR Cancer 2016;2(1):e7. PMID: 28410168
63. Serrano KJ, Coa KI, Yu M, Wolff-Hughes DL, Atienza AA. Characterizing user engagement with health app data: a data mining approach. Translational Behavioral Medicine 2017;7(2):277-85. PMID: 28616846
64. Sharpe EE, Karasouli E, Meyer C. Examining Factors of Engagement With Digital Interventions for Weight Management: Rapid Review. JMIR Research Protocols 2017;6(10):e205. PMID: 29061557
65. Smahel D, Elavsky S, Machackova H. Functions of mHealth applications: A user's perspective. Health Informatics Journal 2017:1460458217740725. PMID: 29121831

66. Solbrig L, Jones R, Kavanagh D, May J, Parkin T, Andrade J. People trying to lose weight dislike calorie counting apps and want motivational support to help them achieve their goals. Internet Interventions 2017;7:23-31. PMID: 28286739
67. Struik LL, Bottorff JL, Baskerville NB, Oliffe JL. The Crush the Crave Quit Smoking App and Young Adult Smokers: Qualitative Case Study of Affordances. JMIR MHealth and UHealth 2018;6(6):e134. PMID: 29884602
68. Sun L, Wang Y, Greene B, et al. Facilitators and barriers to using physical activity smartphone apps among Chinese patients with chronic diseases. BMC medical informatics and decision making 2017;17(1):44. PMID: 28420355
69. Switsers L, Dauwe A, Vanhoudt A, Van Dyck H, Lombaerts K, Oldenburg J. Users' Perspectives on mHealth Self-Management of Bipolar Disorder: Qualitative Focus Group Study. JMIR MHealth and UHealth 2018;6(5):e108. PMID: 29720363
70. Taki S, Russell CG, Lymer S, et al. A Mixed Methods Study to Explore the Effects of Program Design Elements and Participant Characteristics on Parents' Engagement With an mHealth Program to Promote Healthy Infant Feeding: The Growing Healthy Program.
71. Tang J, Abraham C, Stamp E, Greaves C. How can weight-loss app designers' best engage and support users? A qualitative investigation. British journal of health psychology 2015;20(1):151-71 doi: 10.1111/bjhp.12114. PMID: 25130682
72. Tudor-Sfetea C, Rabee R, Najim M, et al. Evaluation of Two Mobile Health Apps in the Context of Smoking Cessation: Qualitative Study of Cognitive Behavioral Therapy (CBT) Versus Non-CBT-Based Digital Solutions. JMIR MHealth and UHealth 2018;6(4):e98. PMID: 29669708
73. Wang N, Deng Z, Wen LM, Ding Y, He G. Understanding the Use of Smartphone Apps for Health Information Among Pregnant Chinese Women: Mixed Methods Study. JMIR Mhealth Uhealth 2019;7(6):e12631 doi: 10.2196/12631. PMID: 31215516
74. Webcredible. Presentation of their findings on digital healthcare for Public Health England (PHE). Unpublished., 2016.
75. Woldaregay AZ, Issom DZ, Henriksen A, et al. Motivational Factors for User Engagement with mHealth Apps. Studies in health technology and informatics 2018;249:151-57. PMID: 29866972
76. Xie Z, Nacioglu A, Or C. Prevalence, Demographic Correlates, and Perceived Impacts of Mobile Health App Use Amongst Chinese Adults: Cross-Sectional Survey Study. JMIR MHealth and UHealth 2018;6(4):e103. PMID: 29699971
77. Zeng EY, Vilardaga R, Heffner JL, Mull KE, Bricker JB. Predictors of Utilization of a Novel Smoking Cessation Smartphone App. Telemedicine journal and e-health : the official journal of the American Telemedicine Association 2015;21(12):998-1004. PMID: 26171733 Frontiers in endocrinology 2019;10:397-97 doi: 10.3389/fendo.2019.00397. PMID: 31293515
