# Supplementary material for: Influences on the Uptake of and Engagement With Health and Well-Being Smartphone Apps: Systematic Review
Source: J Med Internet Res. 2020 May 29;22(5):e17572. doi: 10.2196/17572 (PMC7293059; doi:10.2196/17572)
Supplement: Multimedia Appendix 5 [file jmir_v22i5e17572_app5.pdf]

Multimedia Appendix 5. Critical appraisal of the methodological quality of included studies

1. Qualitative studies

First author	Year	Citation	Q 1.1	Q 1.2.	Q 1.3.	Q 1.4.	Q 1.5.
Anderson	2016		Yes	Yes	Yes	Yes	Yes
Attwood*	2016		Yes	Yes	Yes	Yes	Yes
Baretta	2019		Yes	Yes	Yes	Yes	Yes
Baskerville	2016		Yes	Yes	Yes	Yes	Yes
Casey	2014		Yes	Yes	Yes	Yes	Can't tell
Crane	2017		Yes	Yes	Yes	Yes	Yes
Gorton*	2011		Yes	Yes	Can't tell	No	Yes
Gowin	2015		Yes	Yes	Yes	Yes	Yes
Laurie	2016		Yes	Yes	Yes	Yes	Yes
Lieffers	2018		Yes	Yes	Yes	Can't tell	Yes
Ly	2014		Yes	Yes	Yes	Yes	Yes
Milward	2018		Yes	Yes	Yes	Yes	Yes
Peng	2016a		Yes	Yes	Yes	Yes	Yes
Peng	2016b		Yes	Yes	Yes	Yes	Yes
Perski	2017		Yes	Yes	Yes	Yes	Yes
Perski*	2018		Yes	Yes	Yes	Yes	Yes
Peters	2018		Yes	Yes	Yes	Can't tell	Yes
Pung	2018		Yes	Yes	Yes	Yes	Yes
Puszkiewitz*	2016		Yes	Yes	Yes	Yes	Yes
Solbrig	2016		Yes	Yes	Yes	Yes	Yes
Struik	2018		Yes	Yes	Yes	Yes	Yes
Sun	2017						
Switsers	2018		Yes	Yes	Yes	Yes	Yes
Taki*	2019		Yes	Yes	Yes	Yes	Yes
Tang	2015		Yes	Yes	Yes	Yes	Yes
Tudor-Sfetea	2018		Yes	Yes	Yes	Yes	Yes
Wang*	2018		Yes	Yes	Yes	Yes	Yes
Webcredible*	2016		Yes	Yes	Can't tell	Can't tell	Yes
Woldaregay	2018		Yes	Yes	Yes	Can't tell	Yes

Q 1.1. Is the qualitative approach appropriate to answer the research question?
Q 1.2. Are the qualitative data collection methods adequate to address the research question?
Q 1.3. Are the findings adequately derived from the data?
Q 1.4. Is the interpretation of results sufficiently substantiated by data?
Q 1.5. Is there coherence between qualitative data sources, collection, analysis and interpretation?

2. Randomised control trials

First author	Year	Citation	Q 2.1.	Q 2.2.	Q 2.3.	Q 2.4.	Q 2.5.
Bidergaddi	2018		Yes	Can't tell	Yes	Yes	Yes

Q 2.1. Is randomization appropriately performed?
Q 2.2. Are the groups comparable at baseline?
Q 2.3. Are there complete outcome data?
Q 2.4. Are outcome assessors blinded to the intervention provided?
Q 2.5 Did the participants adhere to the assigned intervention?

3. Non-randomised studies

First author	Year	Citation	Q 3.1.	Q 3.2.	Q 3.3.	Q 3.4.	Q 3.5.
Attwood*	2016		Yes	Yes	Yes	Can't tell	Yes
Bhuyan	2016		Yes	No	Yes	Can't tell	Can't tell
Carroll	2017		Yes	Yes	Yes	Yes	Yes
Guertler	2015		Yes	Yes	Yes	Can't tell	Yes
Puszkiewitz*	2016		Yes	Yes	Yes	Yes	Yes
Serrano	2017		Yes	Yes	Yes	Yes	Yes
Sharpe	2018		Can't tell	Yes	Yes	Can't tell	Yes
Zeng	2015		Yes	Yes	Yes	Yes	Yes

Q 3.1. Are the participants representative of the target population?
Q 3.2. Are measurements appropriate regarding both the outcome and intervention (or exposure)?
Q 3.3. Are there complete outcome data?
Q 3.4. Are the confounders accounted for in the design and analysis?
Q 3.5. During the study period, is the intervention administered (or exposure occurred) as intended?

4. Quantitative descriptive studies

First author	Year	Citation	Q 4.1.	Q 4.2.	Q 4.3.	Q 4.4.	Q 4.5.
Bender	2014		Yes	Yes	Can't tell	Yes	Yes
Gorton*	2011		Yes	Can't tell	Yes	Can't tell	Yes
Mackert	2016		Can't tell	No	Yes	Yes	Yes
Mitchell	2017		Yes	Yes	Yes	Yes	Yes
Perski*	2018		Yes	Can't tell	Yes	Yes	Yes
Smahel	2017		Can't tell	Can't tell	Yes	Can't tell	Yes
Sun	2017		Yes	Yes	Yes	Yes	Yes
Taki*	2019		Yes	Yes	Yes	Can't tell	Can't tell
Wang*	2018		Yes	Yes	Yes	Yes	Yes
Webcredible*	2016		Yes	Yes	Yes	Can't tell	Can't tell
Xie	2018		Can't tell	Yes	Yes	Yes	Yes

Q 4.1. Is the sampling strategy relevant to address the research question?
Q 4.2. Is the sample representative of the target population?
Q 4.3. Are the measurements appropriate?
Q 4.4. Is the risk of nonresponse bias low?
Q 4.5. Is the statistical analysis appropriate to answer the research question?

5. Mixed methods studies

First author	Year	Citation	Q 5.1.	Q 5.2.	Q 5.3.	Q 5.4.	Q 5.5.
Attwood*	2016		Yes	Yes	Yes	Yes	Yes
Gorton*	2011		No	Can't tell	Can't tell	Can't tell	Can't tell
Perski*	2018		Yes	Yes	Yes	Yes	Yes
Puszkiewitz*	2016		Yes	Yes	Yes	Yes	Yes
Taki*	2019		Yes	Yes	Can't tell	Can't tell	Can't tell
Wang*	2018		Yes	Yes	Yes	Yes	Yes
Webcredible*	2016		No	No	Can't tell	Can't tell	Can't tell

Q 5.1. Is there an adequate rationale for using a mixed methods design to address the research question?

Q 5.2. Are the different components of the study effectively integrated to answer the research question?

Q 5.3. Are the outputs of the integration of qualitative and quantitative components adequately interpreted?

Q 5.4. Are divergences and inconsistencies between quantitative and qualitative results adequately addressed?

Q 5.5. Do the different components of the study adhere to the quality criteria of each tradition of the methods involved?

*mixed methods studies. Following the instruction of the MMAT guidance the mixed-methods studies first were assessed on their qualitative and quantitative components independently, and finally using the questions 5.1. – 5.5. on their mixed-methods methodology.

Note: all studies answered 'yes' to the first two screening questions of the MMAT:
S.1. Are there clear research questions?
S.2. Do the collected data allow to address the research questions?
